# An easily implemented single‐visit survey method for intermittently available and imperfectly detectable wildlife applied to the Florida east coast diamondback terrapin (*Malaclemys terrapin tequesta*)

**DOI:** 10.1002/ece3.11130

**Published:** 2024-03-24

**Authors:** Eric D. Stolen, David R. Breininger, Daniel J. Breininger, Robert D. Breininger

**Affiliations:** ^1^ Herndon Solutions Group, LLC NASA Environmental and Medical Contract, Kennedy Space Center Florida USA; ^2^ Department of Mathematics Florida Institute of Technology Melbourne Florida USA

**Keywords:** abundance, aquatic animal, availability bias, Bayesian hierarchical model, density, detection bias, plot survey, single visit survey, temporary unavailability, terrapin

## Abstract

Single‐visit surveys of plots are often used for estimating the abundance of species of conservation concern. Less‐than‐perfect availability and detection of individuals can bias estimates if not properly accounted for. We developed field methods and a Bayesian model that accounts for availability and detection bias during single‐visit visual plot surveys. We used simulated data to test the accuracy of the method under a realistic range of generating parameters and applied the method to Florida's east coast diamondback terrapin in the Indian River Lagoon system, where they were formerly common but have declined in recent decades. Simulations demonstrated that the method produces unbiased abundance estimates under a wide range of conditions that can be expected to occur in such surveys. Using terrapins as an example we show how to include covariates and random effects to improve estimates and learn about species‐habitat relationships. Our method requires only counting individuals during short replicate surveys rather than keeping track of individual identity and is simple to implement in a variety of point count settings when individuals may be temporarily unavailable for observation. We provide examples in R and JAGS for implementing the model and to simulate and evaluate data to validate the application of the method under other study conditions.

## Graphical abstract

We developed an easily implemented single‐visit survey method to estimate the abundance of animals subject to both availability and detection bias. We show using simulations that the method returns reliable parameter estimates. We demonstrate application with eastern diamondback terrapins in Florida, USA, and we provide complete analysis details to allow use of the method for other applications.

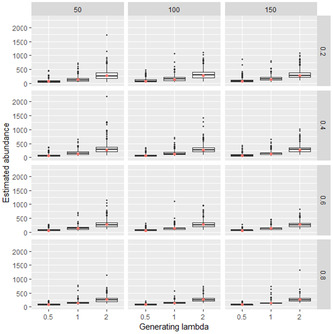

## 1 INTRODUCTION

Understanding spatial patterns in abundance of a wildlife population is a key step in management (Williams et al., 2002). Counts of individuals observed within sample plots during surveys are commonly used to estimate abundance and relate patterns with habitat. However, counts of the number of individuals observed may produce biased abundance estimates due to the inability to detect a constant fraction of all individuals present (Cook & Jacobson, 1979; Kéry & Royle, 2015). In field applications, there are two sources of observation bias: availability bias and detection bias, demonstrated by studies of birds and marine mammals (Farnsworth et al., 2002; Nichols et al., 2009). Availability bias occurs when individuals associated with a sample site are not available for detection, for example when an individual temporarily leaves the sampled area (also known as temporary emigration) or when an individual remains in the sampled area but is in an unobservable state, such as burrowing, not singing, or underwater. It is important to match the scale of sampling to the relative time individuals are not available so that the availability rate is meaningful and can be measured. Detection bias occurs when individuals who are available to be detected are missed due to errors in the detection process. For instance, the rate of visual detection decreases with distance from the observer due to the decreasing apparent size of the object, and to the increasing area sampled as distance increases. Also, human observers may miss objects due to cognitive factors such as fatigue or swamping of attention (Alvarez & Franconeri, 2007; Eckstein et al., 2017). There are also many factors that decrease the detectability of individuals such as behavior, habitat, or environmental conditions; often these can be included as covariates in the detection process (Kéry & Royle, 2015).

Several methods compensate for one or both sources of observation bias. Distance sampling is a proven method for coping with detection bias during single‐visit sampling (Schmidt et al., 2022; Thomas et al., 2014). Double observer methods are an alternative approach that utilizes replicate counts across observers to correct for detection bias (Nichols et al., 2000). Time of detection or removal methods are also used to correct for detection or availability bias (Martin‐Schwarze et al., 2021). When the density within sample plots does not change during a visit, replicate counts can provide additional information to simultaneously estimate detection and availability. A family of models uses correlated replicate counts across time, space, or observers to measure abundance while accounting for one or both sources of observation bias, many of which can be applied during single‐visit surveys (Kéry & Royle, 2015). Single‐visit surveys for abundance employing multiple replicate surveys during the visit are often more efficient when they can provide acceptable accuracy.

Surveys for air‐breathing aquatic animals are an example of sampling in which both intermittent availability and imperfect detection can bias abundance estimates. When animals are below the surface, they are not available to be detected. When animals are at the surface, they may vary in detectability due to environmental conditions (waves and vegetation), methodological factors (plot size, number of observers, and observation platform), or observer factors (fatigue and saturation due to high density). Some previous methods have attempted to measure availability and detection separately and apply a correction factor (Borchers & Cox, 2017; Durden et al., 2011). Other methods have attempted to measure both during single‐visit surveys (Breininger et al., 2019; Martin et al., 2015). The weakness of these methods is keeping track of individuals when not available (e.g., submerged). We present a new method to simultaneously measure detection and availability from replicated counts at closed sites during a brief single‐visit survey. Our method uses distances to individuals to directly estimate detectability and replicate counts of unmarked individuals to estimate abundance and availability. Estimation was done using a hierarchical Bayesian model which allowed a flexible approach for including covariates and hierarchical (random effects) structure.

**FIGURE 1:**
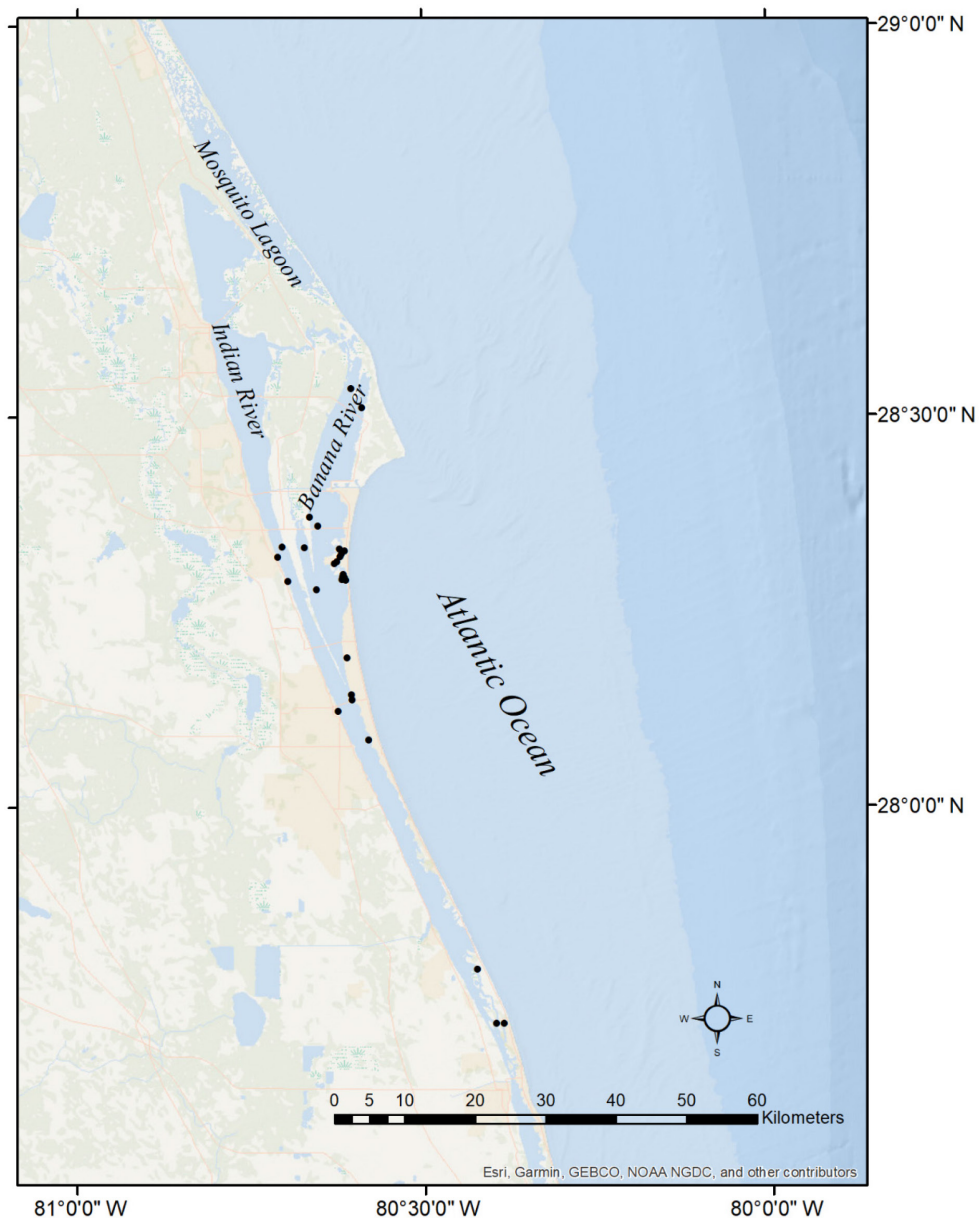
Map of the study site in the Indian River Lagoon system which is composed of the Mosquito Lagoon, the Indian River, and the Banana River on the east coast of Florida, North America. The points show locations of the survey sites.

Our work was motivated by the need to understand the distribution of the Florida east coast terrapin (*Malaclemys terrapin tequesta*), one of seven subspecies of the Diamondback terrapin found in brackish waters along the Atlantic and Gulf coasts of the United States from Massachusetts to Texas. Existing methods to estimate terrapin density based on head counts do not separately estimate detection and availability (Harden et al., 2009; Levasseur et al., 2019). Previously, a survey to estimate terrapin density in the Indian River Lagoon system (IRL, Figure 1) combined distance sampling with removal to account for both detection and availability (Breininger et al., 2019). The survey method in that study required a high degree of observer attention and skill because it required knowing the identity of individuals across replicate samples. We wanted to develop a simple method to survey terrapins that did not require knowing the identity of individuals. While tracking individuals across replicate surveys is often feasible, in other circumstances it may be difficult or impossible. Simplifying the survey method will allow it to be applied by observers with different levels of experience including citizen scientists.

To test our new method, we sampled terrapins in a portion of the IRL and measured four covariates that were expected to influence density (Table  1). We expected low terrapin densities throughout most of the IRL, except near the last remaining locations of breeding, because of long‐term population declines (Breininger et al., 2019; Seigel, 1993). We predicted terrapin densities to be higher near mangroves because terrapins are observed foraging in mangroves in the IRL and other parts of South Florida (Breininger et al., 2019; Denton et al., 2016). We did not include distance to saltmarsh because there was little saltmarsh habitat near the area sampled in this study. We also predicted that terrapins would be more abundant near seagrass beds because they are a likely source of food, terrapins are known to occur in seagrass beds (Lamont et al., 2023), and findings that terrapins have a role in seagrass seed dispersal elsewhere (Tulipani & Lipcius, 2014). We predicted terrapin densities to be greatest near land (mainland or barrier island, but not including dredge spoil islands) because terrapins probably require periodic access to freshwater (Dunson & Mazzotti, 1989) and nesting habitat elevated enough to avoid inundation. We also predicted that terrapin densities would be greater near areas known to be occupied by other terrapins because terrapins often breed in concentrated locations (Seigel, 1980a, 1993). Our objective was to test a method for surveying imperfectly detected and intermittently available animals based on single‐visit surveys with repeated counts within plots. We used the method to learn about factors affecting terrapin density in the IRL, and we discussed how the method could be used to reduce sampling effort to increase the spatial coverage of monitoring surveys across large areas.

**TABLE 1:** Results for the indicator variable model selection approach.

Model	Frequency
Distance to land	0.29
Distance to land + seagrass state	0.17
Distance to land + distance to mangrove	0.13
Distance to land + distance to mangrove + seagrass state	0.09
Distance to land + distance to occupied	0.09
Distance to land + seagrass state + distance to occupied	0.05
Distance to land + distance to mangrove + distance to occupied	0.04
Seagrass state	0.04
Distance to land + distance to mangrove + seagrass state + distance to occupied	0.03
Distance to mangrove + seagrass state	0.02
NULL	0.01
Seagrass state + distance to occupied	0.01
Distance to mangrove + seagrass state + distance to occupied	0.01
Distance to mangrove	0.01
Distance to occupied	0.00
Distance to mangrove + distance to occupied	0.00

*Note*: Frequency is the proportion of Markov chain Monte Carlo (MCMC) iterations in which the set of variables indicated were included as effects on the density rate parameter (*λ*). For example, the top 2 rows of the table indicate that 29% of the iterations included only the distance to land variable, while 17% included both distance to land and the seagrass state variables.

## 2 METHODS

### 2.1 Model specification

We designed our modeling approach to estimate the abundance of animals within circular sample plots during a point count survey subject to detection and availability bias using a hierarchical model with sub‐models for the ecological process (abundance) and the observation processes (detection and availability). The data for this model consists of replicated counts of individuals within plots (the *s* survey sites). At each plot, a number *j* of short replicate counts is conducted, during which the number *n*_*js*_ and the distance from the observer *d*_*i*_ of each individual observed are recorded. The plots are considered to have a maximum radius of *D*_max_ within which observations are recorded, chosen such that the detection at *D*_max_ is still reasonably high, as is standard in distance sampling (Thomas et al., 2014). Plots are considered closed to movement in or out across the replicate counts, although random movement of individuals in or out should not bias the availability measurement since random errors can be expected to cancel (if plots are very small or the density is very low, then this assumption should be verified). The process level of the model specifies a latent (imperfectly observed) abundance of individuals at the survey site *N*_*s*_ as a Poison random variable with rate $\lambda_{s}$:

$$N_{s}\sim \text{Poisson}\left(\lambda_{s}\right)$$

Covariate effects on abundance, such as age class or environmental factors, can be included as a linear model of the rate parameter $\lambda_{s}$ using an appropriate link function:

$$\log\left(\lambda_{s}\right)=\alpha +\mathrm{\beta X}$$

where α is an intercept and *βX* is the matrix multiplication of a parameter vector and design matrix.

For each replicate count, the number of individuals observed is modeled as a binomial count with probability equal to the joint probability of an individual being available and detected during a replicate count period, *p*_*d,s*_. This is equal to the product of the mean probability of detection based on distance *p*_*d,s*_, and the probability of availability during a count *p*_*a,s*_, estimated based on information in the correlated replicate counts, as in a standard N‐mixture model (Kéry & Royle, 2015):

$$y_{\mathrm{sj}}\sim \text{Binomial}\left(p_{t,s},N_{s}\right)$$

$$p_{t,s}=p_{d,s}\cdot p_{a,s}$$

The distance sampling estimation can use any of the available distance detection functions (Kéry & Royle, 2015). To illustrate we used a binned half‐normal detection function:

$$p_{d,s}=\sum_{B}e^{\frac{-{M_{b}}^{2}}{\left(2\sigma^{2}\right)}}$$

where *M*_*b*_ is the midpoint of bin *b*, *σ* is the scale parameter of the detection function, and the summation is over an arbitrary set of distance bins *B*. The number of bins should be large to avoid numerical integration error (Kéry & Royle, 2015). As with the abundance rate parameter, covariate effects on detection can be included using a linear model of *σ* as in standard distance sampling (see Breininger et al., 2019 for an example):

$$\log\left(\sigma \right)=\alpha +\mathrm{\beta X}$$

Assumptions of this component include those for distance sampling including perfect detection at zero distance, no measurement error, and no evasive movement (Thomas et al., 2014). Similarly, covariate effects on availability can also be included using a logit (or other suitable) link function:

$$\text{logit}\left(p_{a,s}\right)=\alpha +\mathrm{\beta X}$$

Further assumptions of our model include those for standard N‐mixture modeling including population closure between replicate surveys and the absence of unmodeled heterogeneity in detection and availability (Kéry & Royle, 2015).

### 2.2 Simulation study

We performed a simulation study to explore the performance of the model at estimating abundance and detection parameters, and to determine the range of parameter space under which the model could correctly estimate the generating values. Data was simulated to replicate a survey with *s* = 25, 50, 100, or 200 sample plots, each with maximum detection distance of 100 m, and *J* = 3 replicate counts of equal length. The number of individuals at sample plots ($\lambda_{s}$) were generated as random draws from a Poisson distribution with the mean selected from (0.5, 1, 2) based on densities observed in a previous study (Breininger et al., 2019). The availability of individuals *p*_*a*_ was constant for all individuals during each replicate count period and was chosen from (0.2, 0.4, 0.6, 0.8). The probability of detection of simulated individuals *p*_*d*_ was determined based on their simulated distance, which was based on choosing a random location for all individuals available for detection during each replicate survey period. The probability of detection was calculated with the half‐normal detection function with *σ* (the scale parameter in the detection function) chosen from (50, 100, 150). The average detection of individuals averaged across all distances for the three levels of *σ* was 0.43, 0.79, and 0.90, respectively, to represent a range of detections including below and above that observed in a previous study (Breininger et al., 2019). We included covariates that affected the availability, detection, and abundance, each generated as random normal variates with mean of 0 and standard deviation of 1, as would typically be used in practice after transforming covariates. When simulating each data set, we used effect sizes (beta parameters) of 1.0 for availability, −1.0 for detection, and 1.0 and −1.0 for the two covariates affecting lambda.

For each of the 144 combinations of number of *s*, *λ*_*s*_, *p*_*a*_, and *σ* (4*3*4*3 = 144 parameter sets) we generated 500 data sets, resulting in 72,000 total data sets (144*500) and fit the data to the model using JAGS 4.3.0 (Plummer, 2017) implemented in R (R Core Team, 2023) with the package jagsUI (Kellner, 2021). For each analysis, we ran an adaptation phase of up to 10,000 iterations (decided automatically by JAGS), then sampled in blocks of 2500 iterations until the Gelman‐Rubin convergence diagnostic (*R*‐hat) for all parameters was <1.05, or a maximum of 300,000 iterations occurred. Posterior estimates were based on the final block of iterations, discarding all previous iterations as burn‐in. For data sets that did not reach convergence, we noted the failure but discarded the results. The details for the simulation approach are available in Supplementary Methods.

For each parameter, we calculated the Markov Chain Monte Carlo (MCMC) convergence rate, and the 95% credible interval (CI) coverage (the proportion of iterations in which the credible interval contained the value used to simulate the data). Bias was measured as the mean relative error: $\frac{1}{n}\sum_{1}^{n}\left(E_{i}-G\right)/G$, and precision was assessed by calculating the standard deviation:$\sqrt{\frac{1}{n}\sum_{1}^{n}{\left(E_{i}-\bar{E}\right)}^{2}}$, where *E*_*i*_ was the estimate in *i*th iteration, $\bar{E}$ the mean parameter estimate, and *G* the generating value.

### 2.3 Eastern diamondback terrapin survey

#### 2.3.1 Study system

The IRL, which makes up much of the subspecies' range along the east‐central Florida coast, is about 251 km long and usually <8 km wide, and consists of shallow lagoons (mostly 0.0–3.0 m in depth) with little tidal influence but high biological diversity (Swain et al., 1995). Most of the IRL shoreline has become urbanized since the 1950s except for large barrier island complexes (>60,000 ha) dedicated to the space program and conservation (Duncan et al., 2004). In the last 2 decades the IRL has experienced harmful algae blooms, with associated seagrass die‐offs and fish kills (Breininger et al., 2016; Phlips et al., 2020; Tiling & Proffitt, 2017) creating adverse conditions that might affect terrapins. In addition, drowning in blue crab traps is an issue for diamondback terrapin populations elsewhere (Dorcas et al., 2007) and likely also in the IRL which has an active blue crab fishery (Steele & Bert, 1998).

#### 2.3.2 Field procedures

We established 11 routes of 15 sample plots, each plot separated by 150–300 m, from the northern Indian River terminus, south 140 km to Vero Beach (Figure 1). Since a primary purpose of the survey was to test our new method, we chose locations likely to be suitable terrapin habitats. Each route was in the vicinity of a location where terrapins had been previously sighted by biologists or captured in fish seine nets by the State of Florida Fisheries Monitoring Program (Tremain & Adams, 1995). Routes were surveyed using a 7 m center console boat with a 150 hp engine between April 1 and October 16, 2015. Surveys were conducted on sunny days between 08:30 and 10:30 AM with little or no wind (<5 mph) with the same two to three observers, all of whom were trained to use distance sampling with terrapins in the IRL during a previous study (Breininger et al., 2019). At each sample plot observers tallied the number of terrapins observed during five consecutive 1 min replicate counts. Counts began 1–2 min after arriving at a plot. Observers stood in the center of the boat and scanned 360 degrees using unaided vision recording straight line distances (m) of each terrapin observed within 100 m. Distances were truncated at 60 m during analysis resulting in 1.13 ha plots. Rangefinders were used to calibrate the observer's distance estimation by checking a few estimated distances following sampling. We generally observed terrapins when they were surfacing for air, although a few individuals were observed basking at the surface. After a plot was completed, we quickly traveled to the next plot to decrease the likelihood that terrapins counted in the previous plot could travel to an adjacent plot before it was surveyed.

### 2.4 Data analysis

We analyzed our terrapin survey data using the new model and included four covariates on the abundance rate parameter. Distance to land (D_land) was the distance in meters from the survey center point to the nearest mainland excluding islands. Distance to mangrove (D_Mangrove) was the distance in meters from the survey center point to the nearest area with 0.5 ha of mangrove. Distance to occupied habitat (D_occ) was the distance in meters from the survey center point to the nearest area reported to have terrapins by regional biologists (i.e., Florida Fish and Wildlife Conservation Commission, US Fish and Wildlife Service, Florida Department of Environmental Protection, Florida Institute of Technology, University of Central Florida, East Coast Zoological Society). The presence of seagrass was a categorical variable recording the presence/absence of seagrass within sample sites determined using aerial photos (Breininger et al., 2016; Morris et al., 2022). This variable had three levels based on the condition of seagrass within each 100 m radius site: 3 if the site had seagrass in 2015, 2 if the site had seagrass in 2009 but not in 2015, and 1 if the site did not have seagrass in either year. There were no sites with seagrass in 2015 that did not have seagrass in 2009 due to the widespread decline in seagrass in the IRL during this time (Morris et al., 2022). We scaled the continuous covariates by subtracting the mean and dividing by 2 standard deviations; this puts the regression coefficients for numerical and categorical covariates on similar scales allowing comparison of effect sizes (Gelman, 2008).

To account for unmodeled effects associated with the field conditions (survey dates and the survey locations chosen), we included a group‐level (random) effect of the survey site on terrapin density (*λ*). We also included an “individual level random effect” with a level for each replicate survey, to help account for non‐independence between counts (Harrison, 2014). Overdispersion is a concern in models of counts of individuals when there is a possibility of non‐independence between individuals. Both random effects were given a normal distribution with mean 0 and precision = 0.2 (SD = 2.23) as a prior distribution (Gelman et al., 2008).

To evaluate evidence for covariate effects we used an indicator variable model selection approach (Kuo & Mallick, 1998). We included latent binary indicator variables for the beta parameters for each of the covariates in the linear formula for abundance. These indicator variables were specified as independent binary variables with Bernoulli (0.5) priors. Each binary indicator variable was multiplied by the beta parameter in the linear formula, and thereby determined if the covariate was included or excluded during a particular MCMC iteration. To ensure that the resulting beta parameters were not overly prone to omission due to the MCMC getting stuck within unlikely regions of the parameter space, we used a spike‐and‐slab prior approach (Mitchell & Beauchamp, 1988). We first fit the full model with all covariates included to estimate the mean and standard deviation of each beta parameter when included. We then used normal prior distributions with the estimated means and standard deviations for the spikes, and the diffuse Bernoulli (0.5) priors for the slabs, with the indicator variables determining which portion of the prior was included in the likelihood. This had the effect of keeping the MCMC chains in reasonable regions of parameter space when particular variables were excluded from the model. To evaluate model support we tabulated the number of times each linear combination of variables was included in an MCMC iteration, with the resulting proportions providing relative model support (Royle et al., 2013).

We implemented the hierarchical Bayesian models using JAGS 4.3.0 (Plummer, 2017) implemented in R (R Core Team, 2022) with the package jagsUI (Kellner, 2021). For each analysis, we ran an adaptation phase of up to 10,000 iterations decided automatically by JAGS, and then discarded samples as burn‐in until Gelman‐Rubin convergence diagnostic (*R*‐hat) was <1.01 and the number of effective samples was estimated to be >4000 for all parameters, except for the standard deviation parameters of the random effects. We did not thin the MCMC posteriors (Link & Eaton, 2012). We tested the model goodness of fit using a Bayesian *p*‐value approach that compared the fit of the observed data to the equivalent fit of data simulated under the model with estimated parameters (Kéry & Royle, 2015).

## 3 RESULTS

### 3.1 Simulations

**FIGURE 2:**
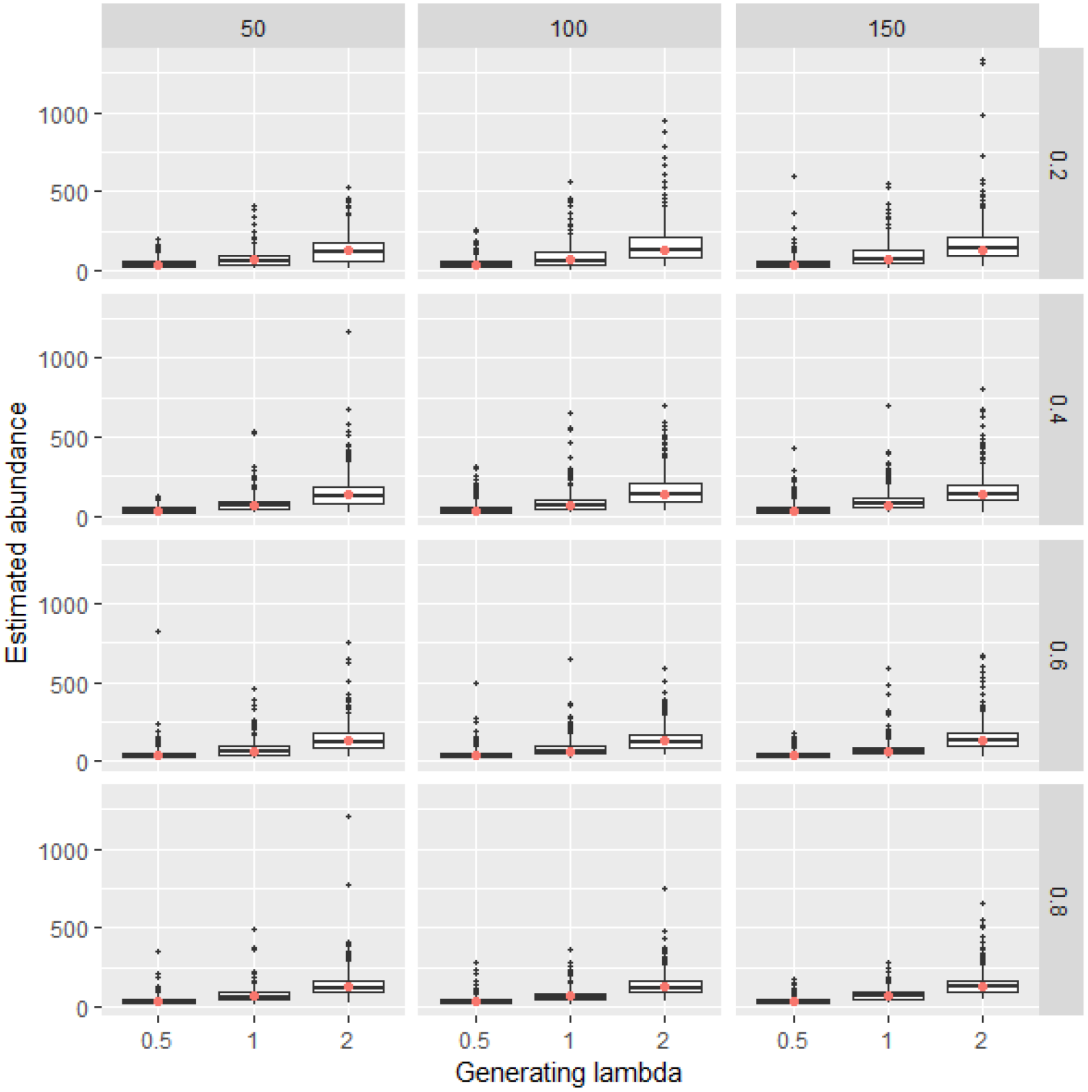
Estimated abundance for 500 simulated data sets at each combination of generating density (lambda, boxes along *x* axis within each panel), availability (constant along rows), and detection (constant along columns) with 25 sampling points. For each box, lower and upper hinges correspond to the inter‐quartile range (IQR), the whiskers extend from the hinges to the most distant value beyond 1.5*IQR from the hinge, and values beyond the whiskers are plotted individually. Simulations in which the Bayesian model did not converge were excluded from the calculation. The red dot is the abundance of the simulated data.

**FIGURE 3:**
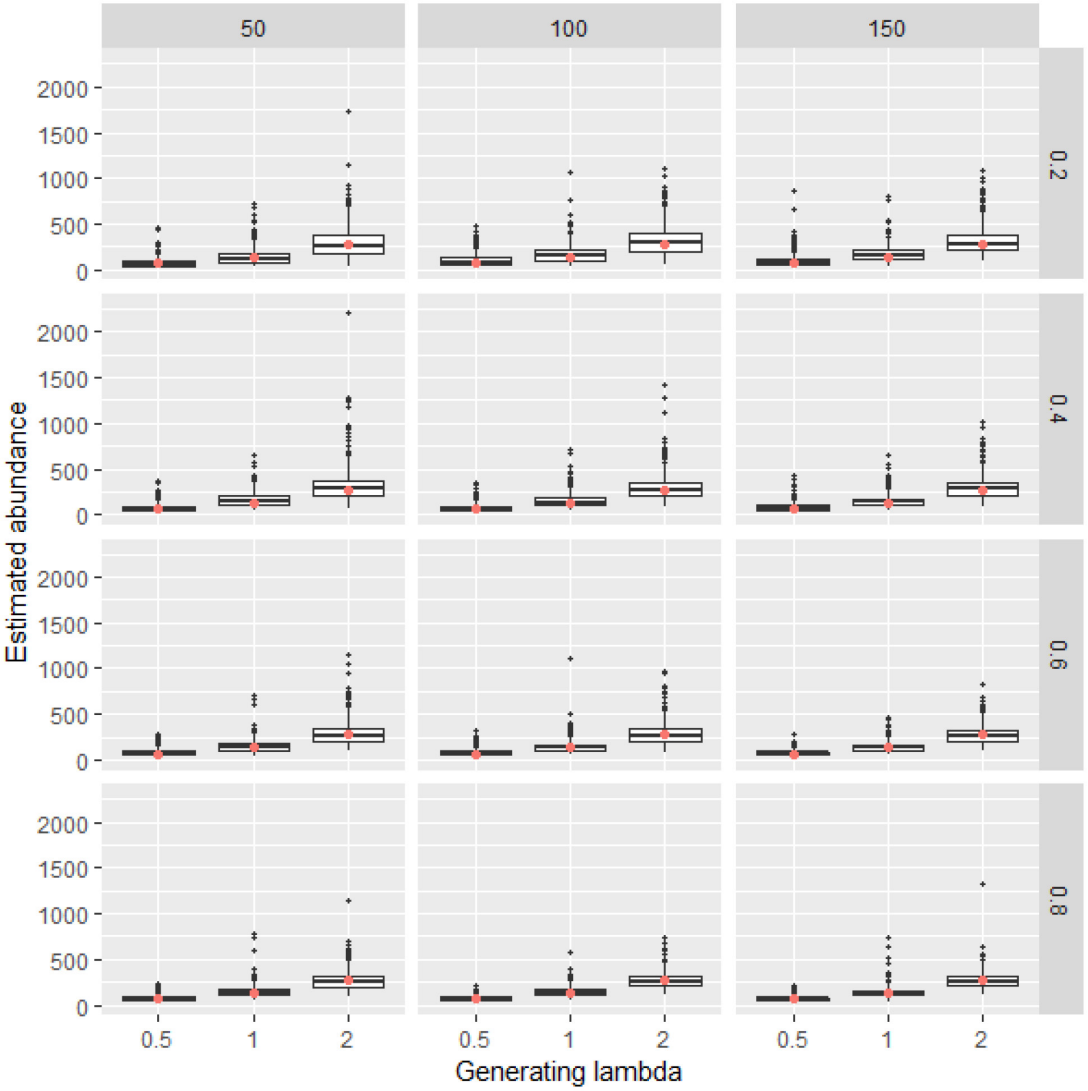
Estimated abundance for 500 simulated data sets at each combination of generating density (lambda, boxes along *x* axis within each panel), availability (constant along rows), and detection (constant along columns) with 50 sampling points. For each box, lower and upper hinges correspond to the inter‐quartile range (IQR), the whiskers extend from the hinges to the most distant value beyond 1.5*IQR from the hinge, and values beyond the whiskers are plotted individually. Simulations in which the Bayesian model did not converge were excluded from the calculation. The red dot is the abundance of the simulated data.

**FIGURE 4:**
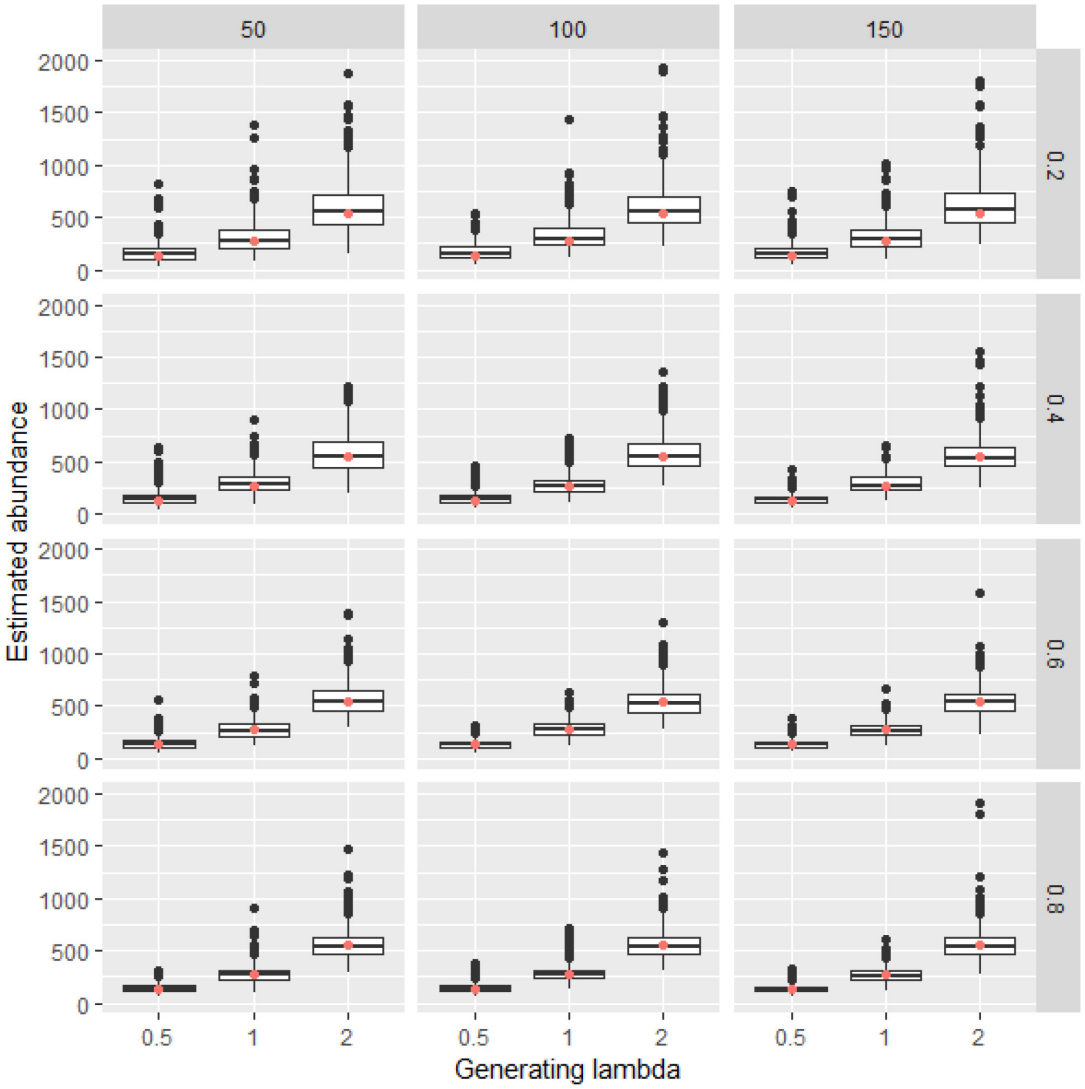
Estimated abundance for 500 simulated data sets at each combination of generating density (lambda, boxes along *x* axis within each panel), availability (constant along rows), and detection (constant along columns) with 100 sampling points. For each box, lower and upper hinges correspond to the inter‐quartile range (IQR), the whiskers extend from the hinges to the most distant value beyond 1.5*IQR from the hinge, and values beyond the whiskers are plotted individually. Simulations in which the Bayesian model did not converge were excluded from the calculation. The red dot is the abundance of the simulated data.

**FIGURE 5:**
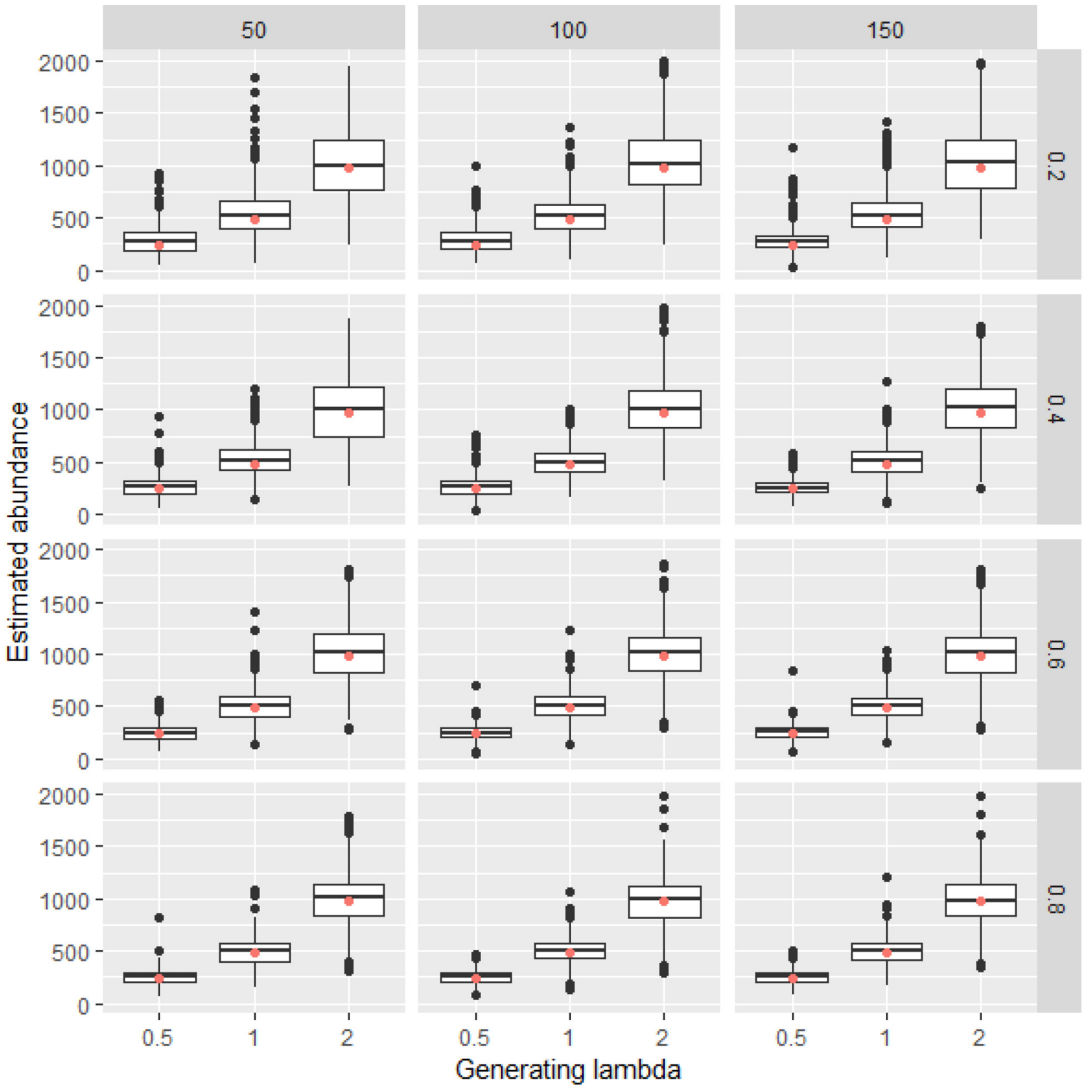
Estimated abundance for 500 simulated data sets at each combination of generating density (lambda, boxes along x axis within each panel), availability (constant along rows), and detection (constant along columns) with 200 sampling points. For each box, lower and upper hinges correspond to the inter‐quartile range (IQR), the whiskers extend from the hinges to the most distant value beyond 1.5*IQR from the hinge, and values beyond the whiskers are plotted individually. Simulations in which the Bayesian model did not converge were excluded from the calculation. The red dot is the abundance of the simulated data.

**FIGURE 6:**
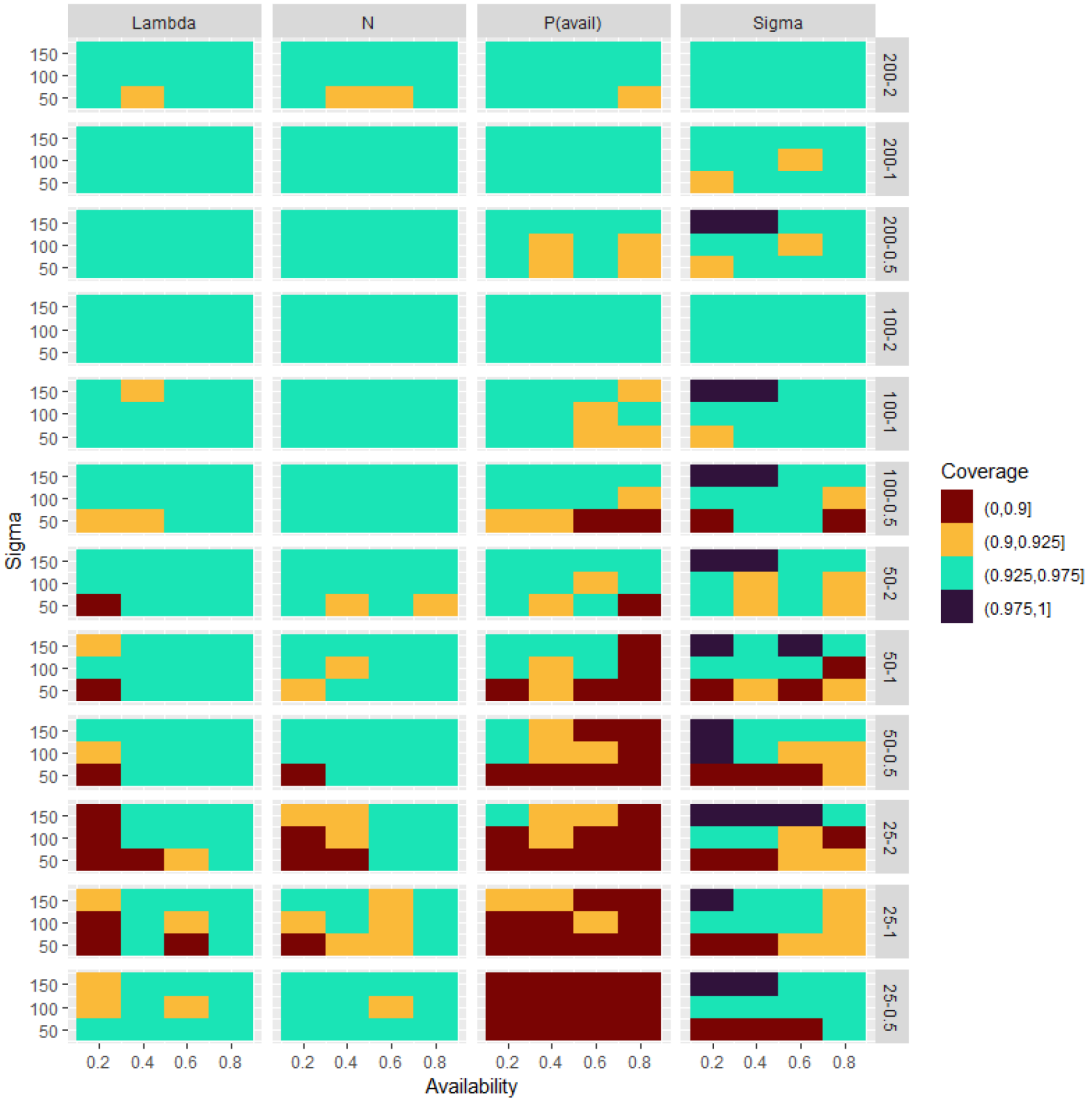
Coverage (proportion of simulations in which the 95% credible interval contained the generating parameter value) for the state parameters (panels along *x*‐axis) from 500 simulated data sets at each combination of number of sample sites and generating density (panels along *y*‐axis), availability (*y*‐axis within panels), and detection (sigma, *x*‐axis within panels). For each panel, the heatmap shows the coverage rate for the 500 simulations for each of the 12 combinations of availability and detection. Sampling conditions improve going up (higher detection within panels, more sites in the top 3 rows of panels) and to the right within panels (higher availability).

**FIGURE 7:**
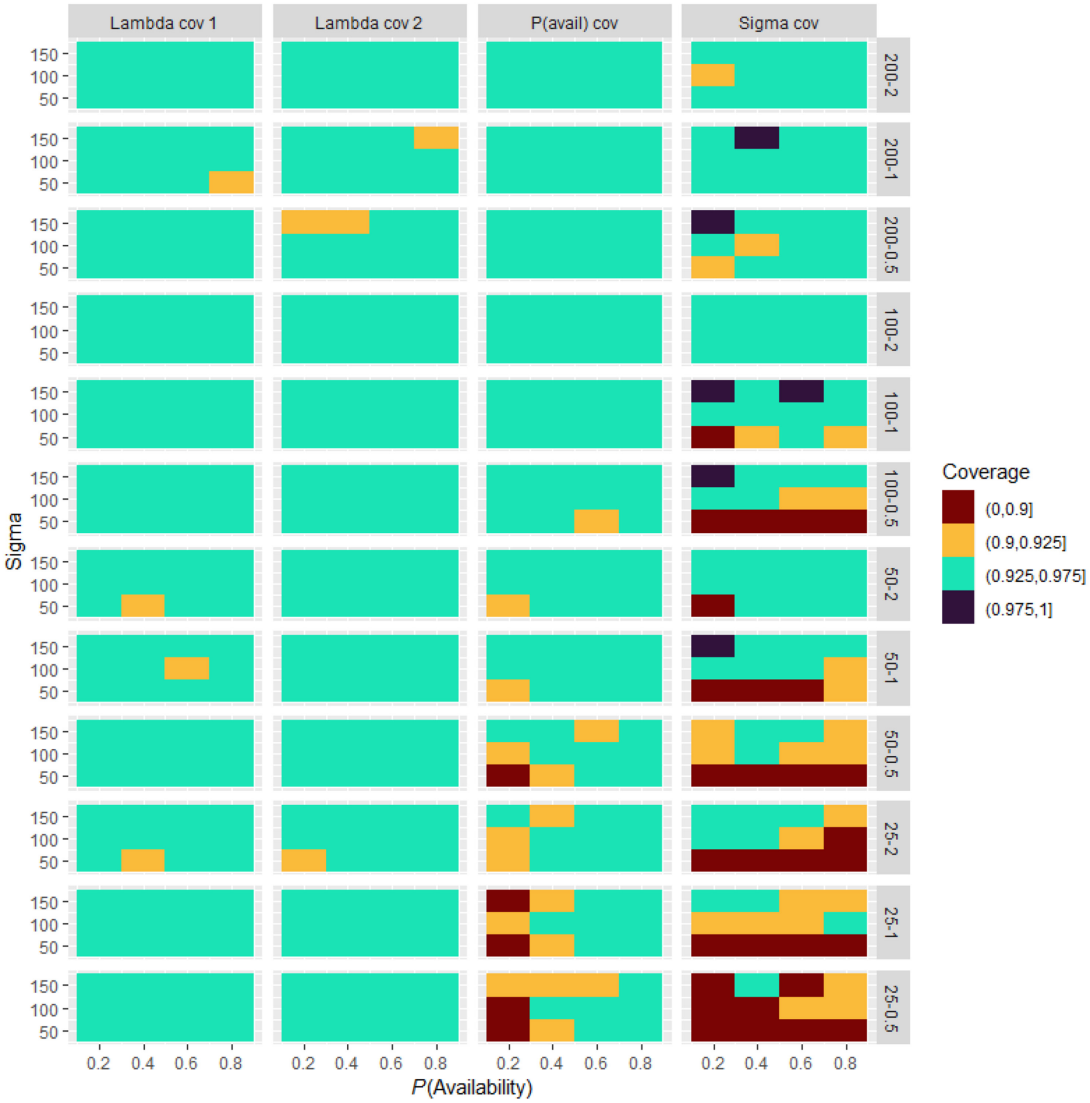
Coverage (proportion of simulations in which the CI contained the generating parameter value) for the state covariate effects (panels along *x*‐axis) from 500 simulated data sets at each combination of number of sample sites and generating density (panels along y‐axis), availability (*y*‐axis within panels), and detection (sigma, *x*‐axis within panels). For each panel, the heatmap shows the coverage rate for the 500 simulations for each of the 12 combinations of availability and detection. Sampling conditions improve going up (higher detection within panels, more sites in the top 3 rows of panels) and to the right within panels (higher availability).

Analysis of simulated data sets showed that the method was able to produce reliable estimates over a wide range of parameter space. Abundance estimates for simulated data sets were unbiased under a wide variety of generating parameter values even at the lowest sample sizes (Figures 2, 3, 4, 5). Likewise, coverage probabilities of 95% credible intervals were near the expected 0.95 level for both the state variables (Figure 6) and the estimated effect sizes (Figure 7) when sample size was 50 sites or greater. Although this is a frequentist property, many users intuitively assume that Bayesian credible intervals will contain the true value at the nominal level, so this is a useful condition. Further simulation of expanded parameter space without covariates showed that when either detection or availability probabilities were moderately high (>0.2 for availability, or >0.43 for detection), the bias of estimates from simulations was near 0 for the state parameters (Figures S1–S4). Relative bias of estimates for the covariate coefficients was also low when either detection or availability probabilities were moderately high (Figures S5–S8). The range of the mean relative error from individual simulated data sets decreased as detection or availability improved, and as the simulated density at the sites increased (lambda values of 0.5, 1, or 2). The relative bias was also lower from simulations with 100 or 200 sites than for those with 25 or 50 (compare S1 and S2 with S3 and S4 for state parameters, or S5 and S6 with S7 and S8 for covariate coefficients). The width of 95% credible intervals of abundance estimates decreased as either the detection or availability increased (Figure S9). Convergence of Bayesian parameter estimates was achieved for most simulated data sets within the 300,000 iteration limit used (Figures S6–S9). Convergence was not achieved for a small number of data sets, mostly those with low availability (5% of data sets with availability = 0.2, <1% when availability >0.2).

### 3.2 Terrapin survey

Terrapins were observed at only 31 of the 165 sample plots surveyed (15 sample plots at each of 11 sites) showing the clumped nature of terrapin occurrence in the IRL. A total of 92 terrapins were observed during the replicate samples in 64 clusters ranging from 1 to 7 terrapins (mean group size 1.4). We plotted histograms of distances to identify 60 m as an appropriate truncation distance (Thomas et al., 2014) resulting in plots of 1.13 ha area. Model selection identified the model with only the distance to land covariate as the best model). The model that included both distance to land and the state of seagrass was the next best‐supported model with about half as much support. The posterior estimates for both effects were large (Table 2). The posterior estimates from the full Bayesian model incorporate model selection and thus give a type of model‐averaged prediction of density (Royle et al., 2013). For sites without seagrass in either year, the estimated density of terrapins at 75, 115, and 215 m from land (the 25th, 50th, and 75th percentiles of the distances observed) was 0.21, 0.17, and 0.096 individuals/ha, respectively. At sites with seagrass in both 2009 and 2015, the estimated densities were much lower: 0.079, 0.0061, and 0.0036 individuals/ha, respectively. Neither distance to mangrove nor distance to occupied sites had much support from the model selection procedure, and their estimated coefficients broadly overlapped zero. The estimated mean probability of availability was 0.58 and the mean probability of detection of an individual during a replicate sample was 0.25. The Bayesian model did not show evidence of a lack of fit based on the goodness of fit check (Bayesian *p*‐value .39).

**TABLE 2:** Posterior parameter estimates from the Bayesian model of factors influencing terrapin density using indicator variables to allow selective inclusion of covariates.

Parameter	Predicted effect	Mean	SD	2.50%	97.50%
Abundance intercept		−1.87	0.63	−3.07	−0.65
Distance to mangrove	−	−0.94	0.81	−2.58	0.61
Distance to land	−	−2.70	1.18	−5.17	−0.56
Seagrass 2009 only	+	−0.40	0.71	−1.81	0.99
Seagrass 2009 & 2015	+	−3.34	1.71	−6.97	−0.24
Distance to occupied	+	−0.28	0.53	−1.35	0.73
Include dist. to mangrove		0.33	0.47	0.00	1.00
Include dist. to all land		0.90	0.30	0.00	1.00
Include seagrass		0.42	0.49	0.00	1.00
Include dist. to occupied		0.24	0.43	0.00	1.00
Availability intercept (logit scale)		0.33	0.18	0.07	0.74
Sigma (scale of detection function)		21.62	1.43	19.13	24.73
Survey route RE SD		1.91	0.35	1.32	2.70
Overdispersion RE SD		2.82	1.32	0.82	5.83
Bayesian *p*‐value		0.39	0.49	0.00	1.00

*Note*: Four variables were included in the density model (distance to mangrove, distance to land, seagrass state, and distance to occupied site). For each of these effects, an indicator variable determined if the effect was included during each iteration of the Bayesian model. The detection models for availability and detection (sigma) did not include modeled covariate effects, but availability included a replicate count level random effect to account for potential overdispersion. A separate random effect at the survey route level was included in the abundance model.

## 4 DISCUSSION

Surveys in which individuals are counted in sample plots are a common method to estimate animal abundance. In some settings, unadjusted counts may produce unbiased estimates of abundance (Johnson, 2008). More typically, bias occurs due to detection error and the lack of availability of some individuals during the counting (Cook & Jacobson, 1979; Kery & Schmidt, 2008). A straightforward approach to address observation bias is to use correlated replicate counts to estimate error rates. When there is one source of observation bias, this can be accomplished in a single visit using double observer, or time of observation/removal sampling methods. To estimate both detection error rate and the rate of availability requires additional information to allow parameters to be identified, which can be provided by replicate surveys (Kéry & Royle, 2015). Several attempts have combined distance sampling and removal methods to achieve this, but removal methods usually require that individuals be identified across replicate samples (Amundson et al., 2014; Breininger et al., 2019). In many field applications, this will be difficult due to the temporary unavailability of individuals. We demonstrated the application of a method based on combining distance sampling and N‐mixture modeling that does not require tracking individual identity across replicate counts. This method simplifies the requirements for single‐visit surveys when both detection and availability bias occur.

Simulations showed that our method produced reliable abundance estimates under a wide range of detection and availability, corresponding to surveys for many animals in the field. The method also performed well when the number of sample sites was 50 or greater. The coverage rates for 95% credible intervals were as expected under these conditions showing that the method can be used as a survey tool. This method is suitable for estimating abundance when individuals occupy the sample plot for a time during which the replicate counts can be obtained. However, a low level of random movement of individuals in and out of plots should not be of concern, provided that the plots are not very small or the density is very low (when small changes in numbers could cause large changes in the estimates). The replicate counts must meet the normal requirements of distance sampling, including perfect detection at zero distance, no measurement error, and no evasive movement (Thomas et al., 2014). If any of these assumptions cannot be met, the model could be expanded to accommodate the departure as in other applications of distance sampling (Buckland et al., 2004). While we developed the method to survey for terrapins, the method could be applied to other situations in which individuals occupy a site for a short period of time but alternate between being available and not available for detection. For example, diving birds foraging in patches of prey (Winiarski et al., 2014). Similarly, aquatic mammals such as otters, dolphins, manatee, or aquatic reptiles such as crocodilians, may remain in areas for short time periods allowing replicate counts (Edwards et al., 2021; Williams et al., 2017). Other applications might include aquatic insect larvae that surface for air (Banks et al., 2018), territorial birds that sing in bouts (Thompson et al., 2017), or burrowing animals (Aguzzi et al., 2021; Lim & Wong, 2010).

Based on simulations we recommend that the probability of detection of individuals during replicate counts be greater than 0.3, which corresponds to a scale parameter (sigma) of at least 40 for our case study half‐normal detection function with maximum distance 60. One way to estimate this is to collect some pilot data of detection distances, and then plot the data in a histogram with many distance bins and compare this with simulated data. There are several ways that detection can be improved during distance sampling plot surveys. For example, the use of an elevated platform, adding more observers or using a slower scanning procedure so that all portions of the plot have more observation time. Also restricting the environmental conditions so that they are optimal for viewing the target species is useful and reduces heterogeneity in detection across samples (Buckland et al., 2015). When sources of detection heterogeneity cannot be eliminated, covariates can be included in the detection model (Breininger et al., 2019; Levasseur et al., 2019). Based on our simulations, we recommend that the probability of availability of individuals during replicate counts be >0.2, although this is not as crucial as improving detection. Although more difficult to improve than detection, availability could be raised by surveying animals during periods when they are more available. If availability is found to be low in a particular application of our method, then simulations should be used to verify model performance, or alternative survey methods used. Researchers might also consider adding more sites to increase sample size and improve the performance of the estimators, as seen in our simulations.

Our method assumes that individuals are not counted more than once during each replicate count (this is not to be confused with the recounting of individuals expected in subsequent replicate counts). A potential issue is that if the replicate samples are too long an individual might be recounted within a replicate sample because it moves into a new portion of the survey area (plot) after first being detected. This issue is not unique to our method (Buckland et al., 2015). The best way to minimize this risk is to have a regular pattern of scanning during the replicate counts that covers all areas for the same amount of time. For example, a single observer could slowly rotate at a constant rate while scanning the plot, trying to spend the same amount of time for all distances. Alternately two or more observers could divide the area to be scanned which would reduce the potential for individuals to be counted twice since the entire area would be sampled more quickly. The rate of scanning could be altered based on knowledge of the specific behavior of the target animals. Replicate counts should be done in a short time relative to the length of bouts of availability/non‐availability to minimize the opportunity for double counting. For example, if a diving animal is unavailable for an average of 1 min, then during a 1‐min sample period you would not expect to see individuals surface multiple times.

From simulations, we found some evidence for a positive bias when density within plots was high. We suspect that this was due to the use of a log transformation as the link function in the abundance sub‐model. If this was a problem in a particular application, a potential remedy could be to explore alternative link functions (Ali, 2022; Wiemann et al., 2021). Alternatively, covariates that predict density might be found to improve the abundance model. High densities may also overwhelm observers during surveys, resulting in more detection bias and adding heterogeneity to the modeling of the detection function. A remedy would be to add more observers or to use smaller plots. We recommend that practitioners conduct simulations based on their sampling design to verify that parameters can be estimated within desired precision and without bias for a particular situation. We provide examples in R for simulating and analyzing data from our survey design (Supporting Information). Parameter values for such simulations could be generated from pilot data, values gleaned from the literature, or even good intuition from studies on similar or related species. Simulation is also useful after data is collected to validate survey methods and modeling procedures (Conn et al., 2018).

Finally, we note that our method is focused on correcting for both detection bias and availability bias within replicate counts during a single‐visit survey. There will be situations in which it is possible to jointly estimate the confounded product of detection and availability rates without needing both sources of information (distances and replicate counts). For example, by using only replicate counts occurring over a period during which sites are closed to changes in abundance and analyzing with N‐mixture models. In many cases, it could be more efficient to use alternative methods if bias does not result from unmet assumptions. Our method is an alternative approach when both availability and detection bias are of concern.

### 4.1 Terrapin survey case study

A goal of this study was to develop field methods to efficiently measure open water terrapin densities. Previous experience in the IRL told us that terrapin densities were usually low, that much apparently suitable habitat seemed unoccupied, and that the effective sampling areas of boat‐based surveys were small. Using our more easily implemented field method, we were able to estimate the density of eastern diamondback terrapins in the IRL system at a level of precision similar to those that required tracking of individual identity across replicate plot counts (Breininger et al., 2019). This demonstrates the possibility for future surveys to employ citizen scientist observers to cover a more extensive area. We note that it is important to properly train volunteers to meet all assumptions of the method, especially regarding the Distance sampling component. Expanding the coverage of sampling would provide an opportunity to adaptively test and refine the understanding of species‐habitat relationships. Greater power to resolve these relationships is needed because extant terrapin populations in the IRL are remnants of a declining population, and preliminary measures of habitat effects on density might represent artifacts from earlier conditions. These relationships may also highlight vulnerabilities that can be targeted by management, such as critical breeding habitat (Seigel, 1980b).

We found that terrapin densities declined with increasing distance to land as predicted, a pattern also found in a previous survey (Breininger et al., 2019), although the effect was stronger and measured with more precision in this study. This suggests using a stratified sampling design with most samples concentrated within a few hundred meters of the shoreline. As with other rare species, a two‐phase adaptive sampling approach could be used, with many less‐intensive samples across large areas to locate high‐density sites, followed by fewer, more intensive samples stratified by habitat (Conroy et al., 2008). The broader sampling could be done using the current method and would identify regions of higher and lower density for the next phase. Then more focused methods such as capture‐recapture could be used to generate more precise abundance estimates, and additional demographic parameters such as recruitment, survival, breeding status, and movements (Levasseur et al., 2022). Interpretation of the specific effects found in this study should be done with caution because our sampling may have been biased towards sites with suitable terrapin habitats versus less suitable areas.

We were initially surprised by the finding that terrapin density was negatively correlated with distance to areas with persistent seagrass. This pattern could occur because terrapin prey (blue crabs, mollusks) are more common (or easier to spot and capture) on muddy bottoms than in seagrass. Another possible reason for this pattern is that sites with persistent seagrass in the IRL occur in shallow water (Breininger et al., 2016, 2019). Or, factors not measured in this study could be causing declines in abundance within seagrass habitat in the IRL. This highlights a need for more data on the distribution and habitat preferences of terrapins in the IRL. Quantifying terrapin habitat relationships could lead to the development of maps of terrapin density in the IRL which could be valuable in defining conservation measures such as crab trap excluders to reduce terrapin drowning (Hart & Crowder, 2011). Developing this information will require much effort given the size of IRL lagoon and the low density and detectability of terrapin there. Methods that can be employed consistently by observers with varying levels of training over large areas are needed to maximize efficient sampling.

## 5 CONCLUSION

We demonstrated a method using simple replicated counts that can account for bias in availability and detection in a single visit to sample plots. Simulations showed acceptable performance under realistic survey conditions, and we demonstrated its use to sample Florida's east coast diamondback terrapin density in the IRL. We also showed how modeling of species‐habitat relationships can be investigated over a large area. This method is useful not only for aquatic species but also for circumstances when availability varies over short periods of time. The flexible Bayesian hierarchical modeling approach allows for covariates on detectability and availability, and other model improvements including accounting for overdispersion using random effects.

## AUTHOR CONTRIBUTIONS

**Eric D. Stolen:** Conceptualization (equal); formal analysis (lead); methodology (equal); software (lead); validation (lead); visualization (lead); writing – original draft (lead); writing – review and editing (lead). **David R. Breininger:** Conceptualization (equal); formal analysis (supporting); investigation (lead); methodology (equal); supervision (lead); writing – original draft (supporting); writing – review and editing (supporting). **Robert D. Breininger:** Conceptualization (supporting); data curation (equal); investigation (equal); methodology (supporting); writing – review and editing (supporting). **Daniel J. Breininger:** Conceptualization (supporting); data curation (equal); investigation (equal); methodology (supporting); writing – review and editing (supporting).

## CONFLICT OF INTEREST STATEMENT

The authors declare that there is no conflict of interests.

## Supporting information


Appendix S1

## Data Availability

Data and code are uploaded as a zipped file for review; If accepted the authors will make all data and code available on the Dryad data repository.
