# Supplementary material for: An easily implemented single‐visit survey method for intermittently available and imperfectly detectable wildlife applied to the Florida east coast diamondback terrapin (Malaclemys terrapin tequesta)
Source: Ecol Evol. 2024 Mar 24;14(3):e11130. doi: 10.1002/ece3.11130 (PMC10961479; doi:10.1002/ece3.11130)
Supplement: Supplementary file 1 — Appendix S1 [file ECE3-14-e11130-s001.zip › ece311130-sup-0002-supinfo.docx]

**Supplemental 1. Additional details of simulation results.**


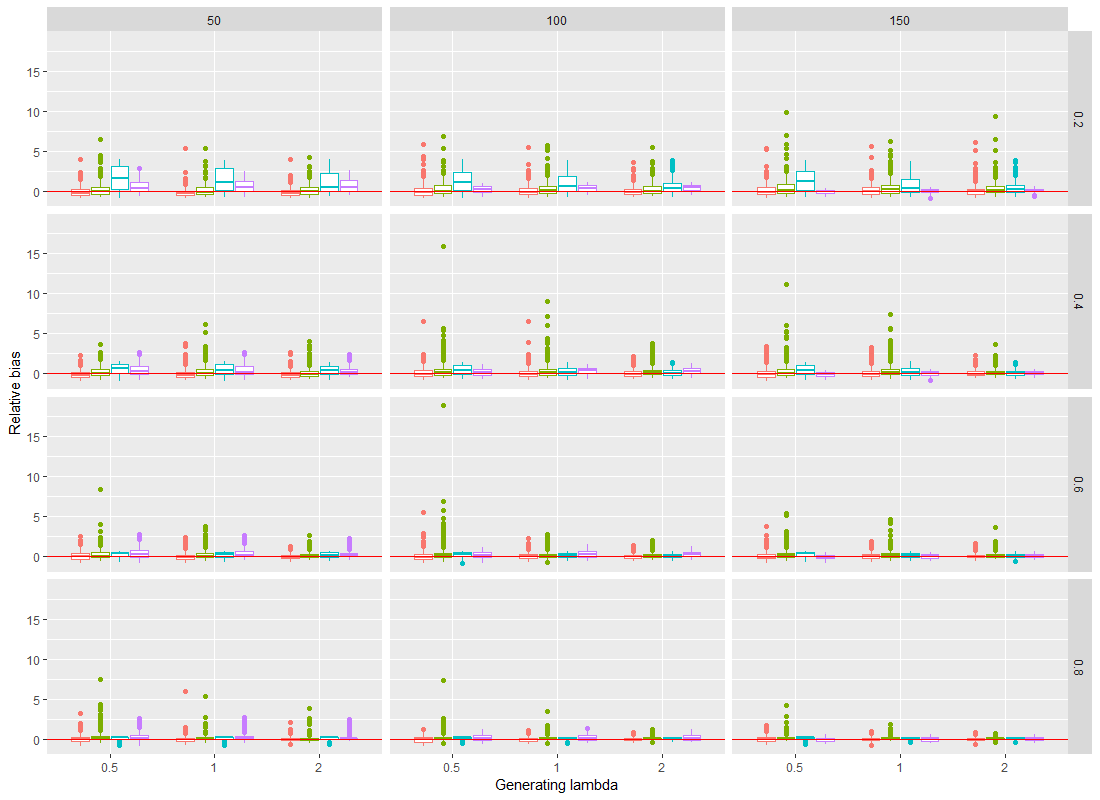


Figure S1. Relative bias for the state parameters in the Bayesian model tested with 500 simulated data sets at each combination of generating density lambda (boxes along x axis within each panel), availability (constant along rows), and detection (constant along columns) with 25 sampling sites. For each level lambda the 4 boxes are for lamdda, N, pa, and sigma.


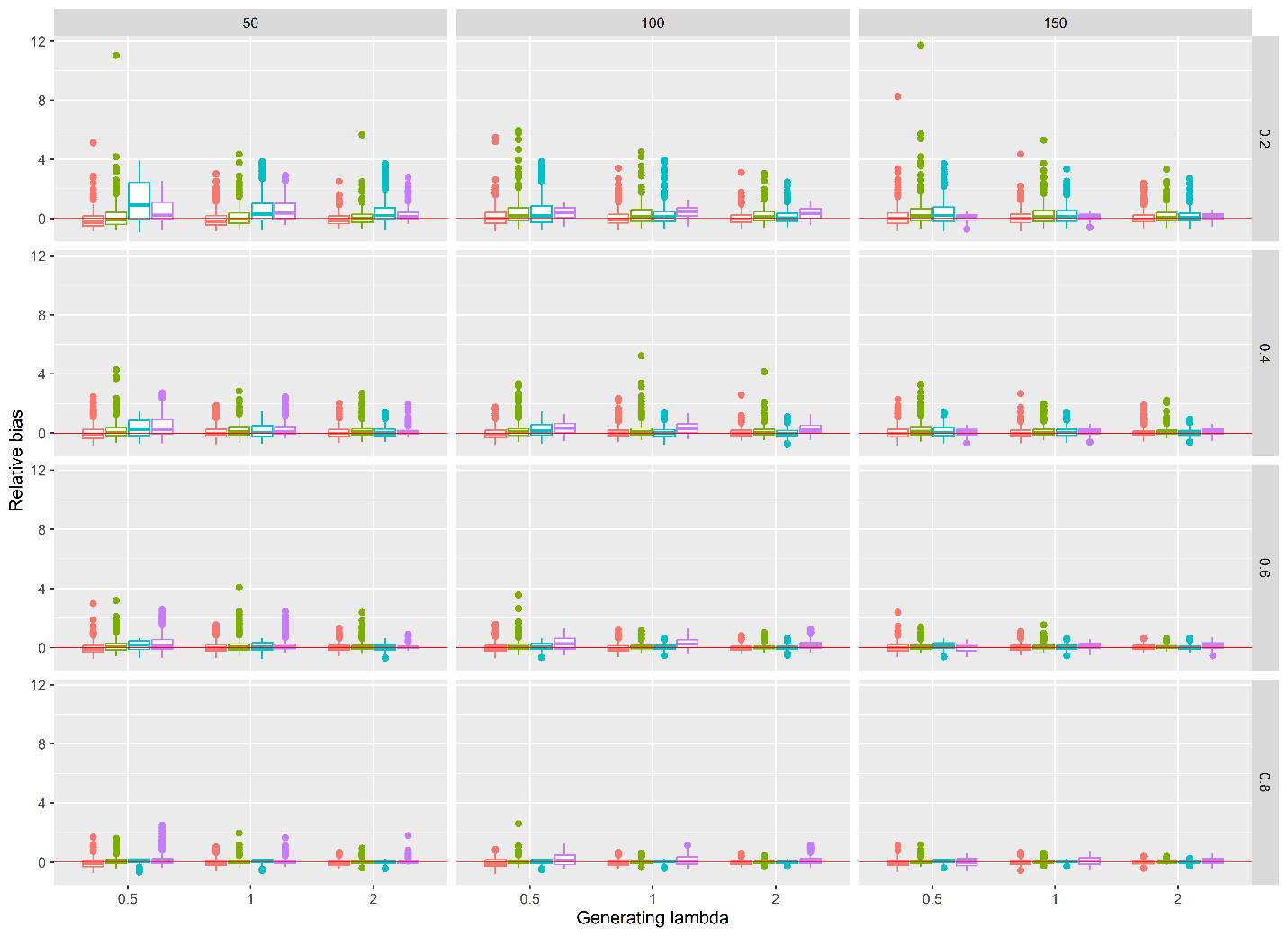

Figure S2. Relative bias for the state parameters in the Bayesian model tested with 500 simulated data sets at each combination of generating density lambda (boxes along x axis within each panel), availability (constant along rows), and detection (constant along columns) with 50 sampling sites. For each level lambda the 4 boxes are for lamdda, N, pa, and sigma.


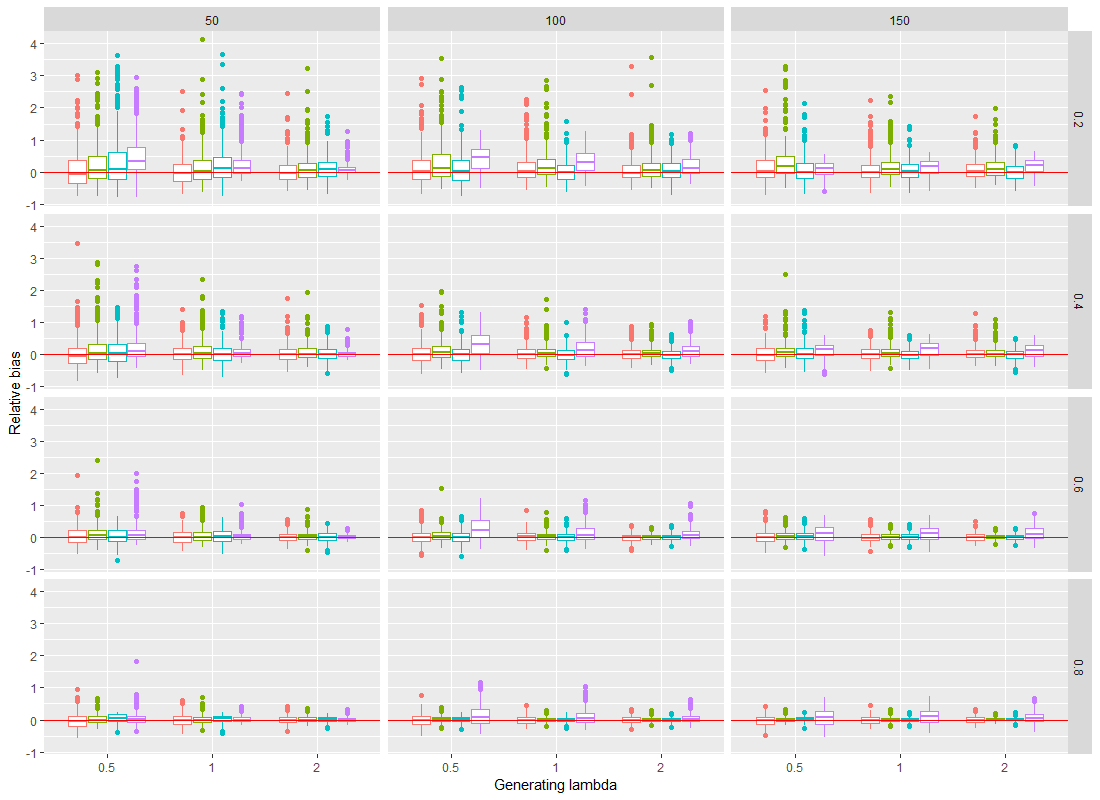


Figure S3. Relative bias for the state parameters in the Bayesian model tested with 500 simulated data sets at each combination of generating density lambda (boxes along x axis within each panel), availability (constant along rows), and detection (constant along columns) with 100 sampling sites. For each level lambda the 4 boxes are for lamdda, N, pa, and sigma.


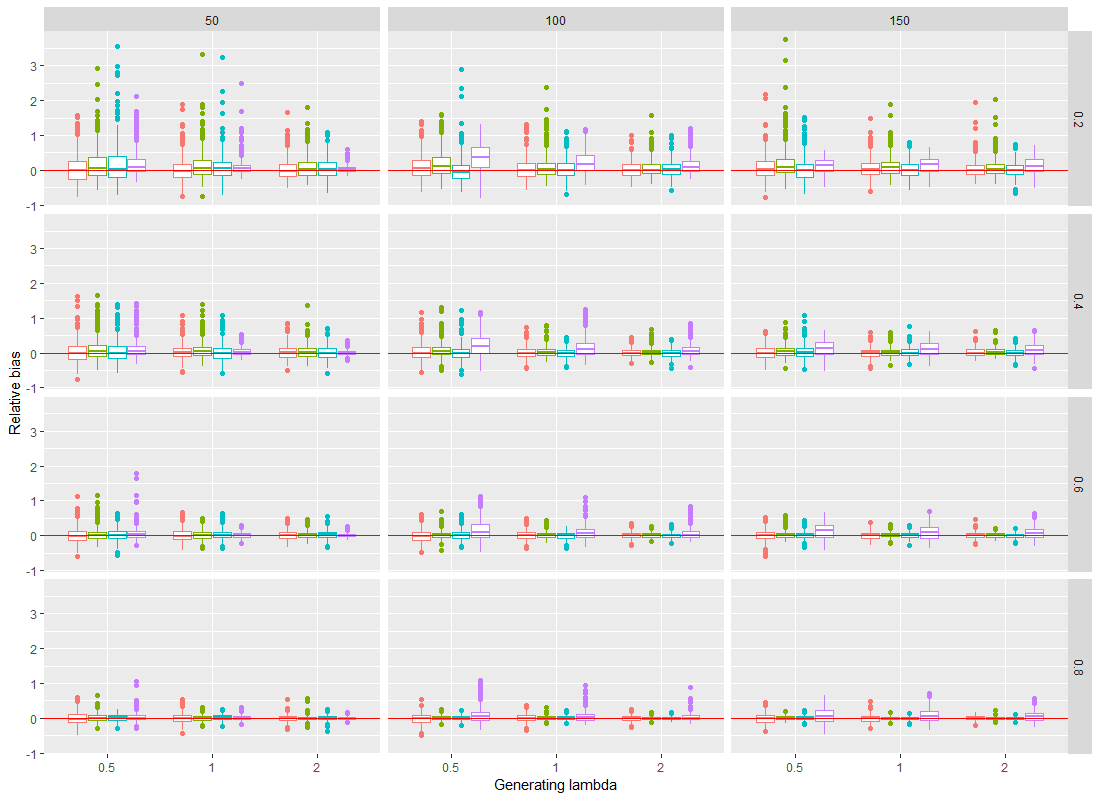


Figure S4. Relative bias for the state parameters in the Bayesian model tested with 500 simulated data sets at each combination of generating density lambda (boxes along x axis within each panel), availability (constant along rows), and detection (constant along columns) with 200 sampling sites. For each level lambda the 4 boxes are for lamdda, N, pa, and sigma.


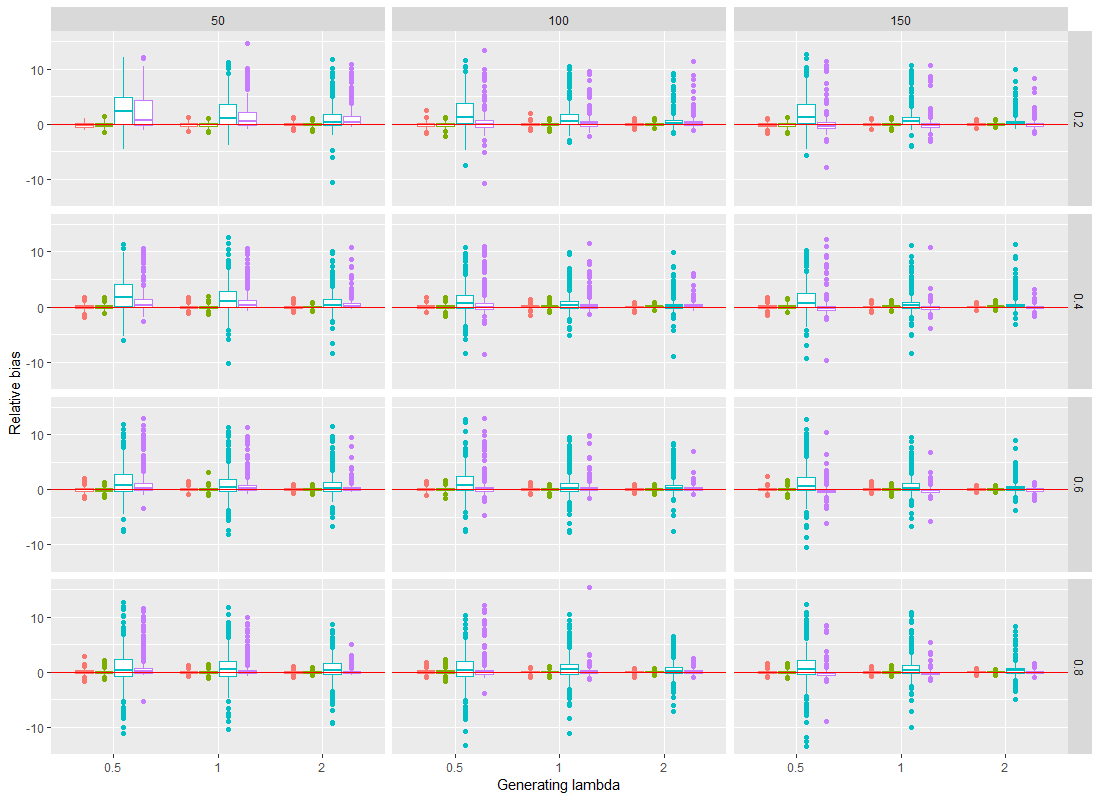
 Figure S5. Relative bias for the estimated effect parameters in the Bayesian model tested with 500 simulated data sets at each combination of generating density lambda (boxes along x axis within each panel), availability (constant along rows), and detection (constant along columns) with 25 sampling sites. For each level lambda the 4 boxes are for the effect of the lamda covariate 1, lambda covariate 2, p.a. covariate, and sigma covariate.


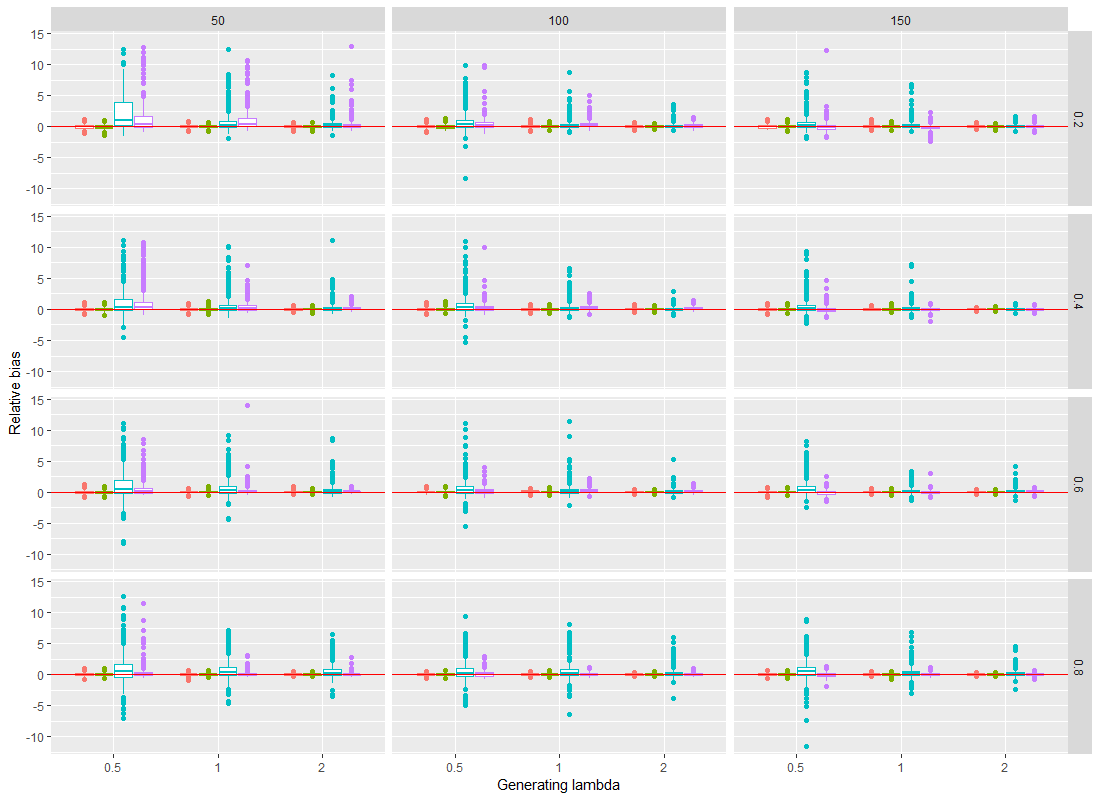


Figure S6. Relative bias for the estimated effect parameters in the Bayesian model tested with 500 simulated data sets at each combination of generating density lambda (boxes along x axis within each panel), availability (constant along rows), and detection (constant along columns) with 50 sampling sites. For each level lambda the 4 boxes are for the effect of the lamda covariate 1, lambda covariate 2, p.a. covariate, and sigma covariate.


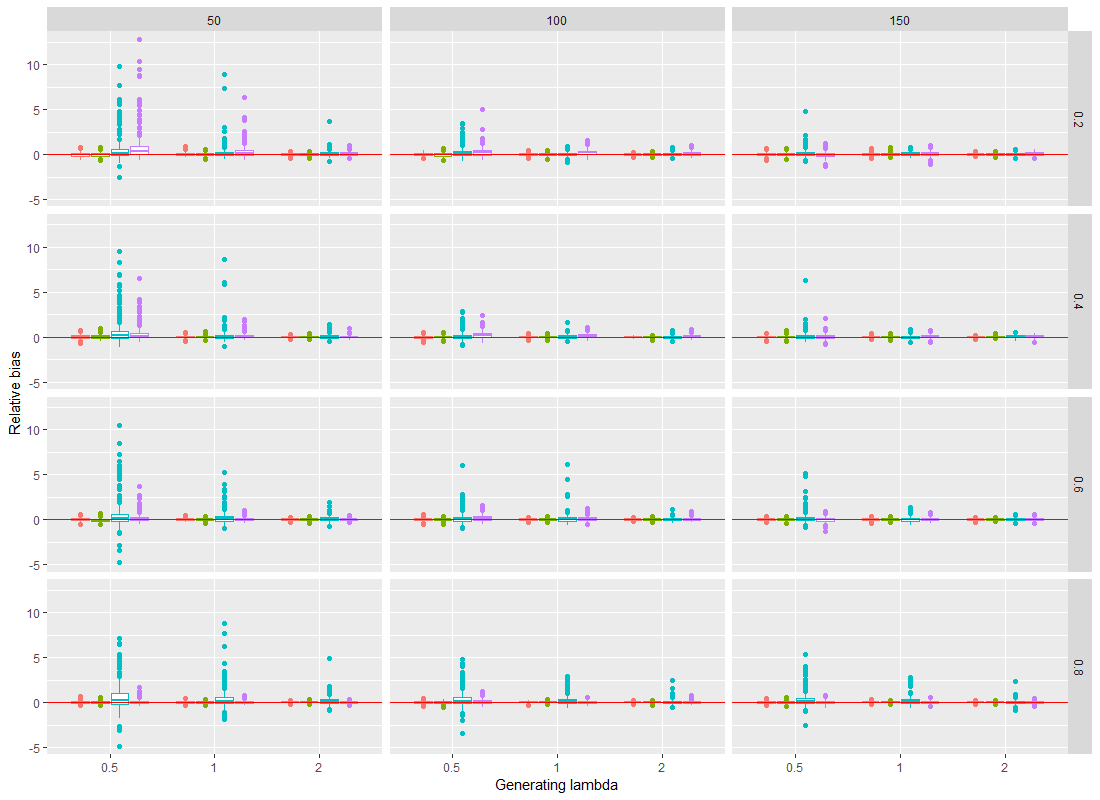


Figure S7. Relative bias for the estimated effect parameters in the Bayesian model tested with 500 simulated data sets at each combination of generating density lambda (boxes along x axis within each panel), availability (constant along rows), and detection (constant along columns) with 100 sampling sites. For each level lambda the 4 boxes are for the effect of the lamda covariate 1, lambda covariate 2, p.a. covariate, and sigma covariate.


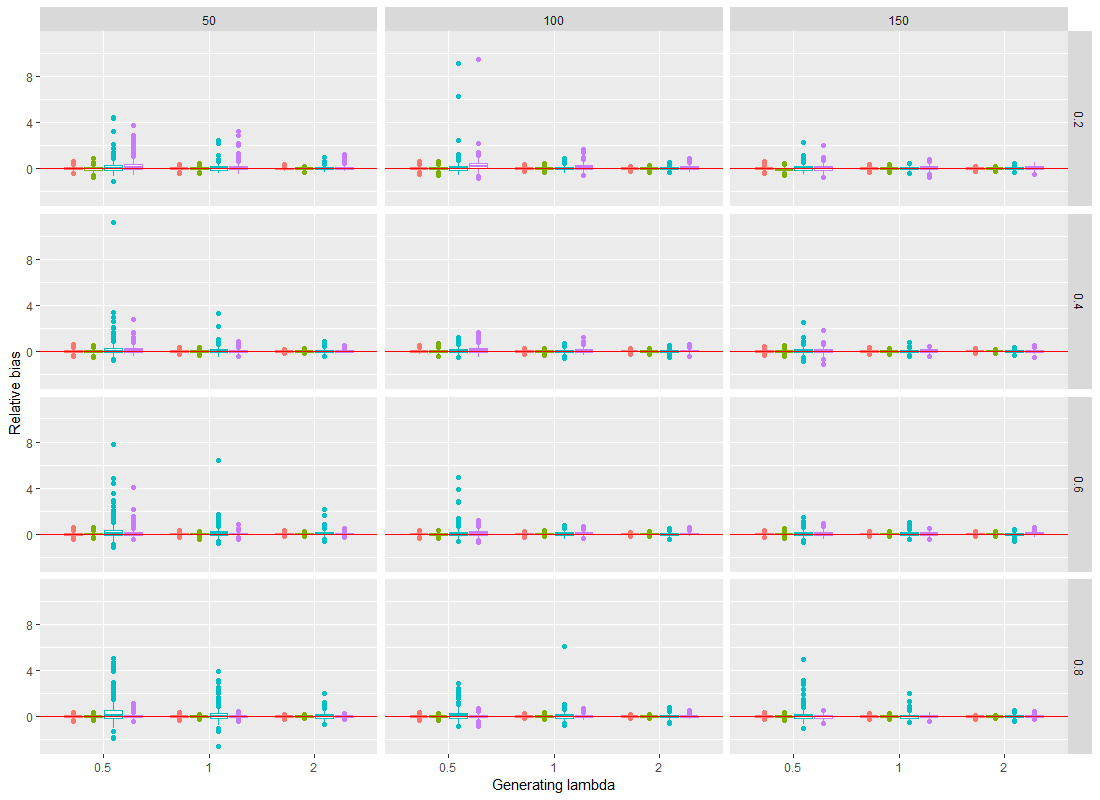


Figure S8. Relative bias for the estimated effect parameters in the Bayesian model tested with 500 simulated data sets at each combination of generating density lambda (boxes along x axis within each panel), availability (constant along rows), and detection (constant along columns) with 200 sampling sites. For each level lambda the 4 boxes are for the effect of the lamda covariate 1, lambda covariate 2, p.a. covariate, and sigma covariate.

**
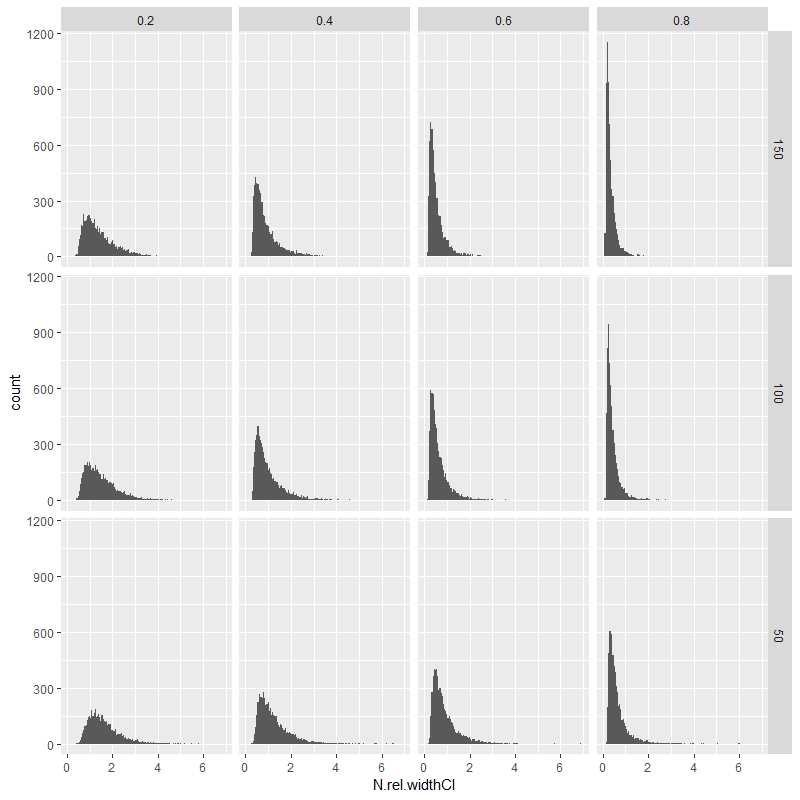
**

Figure S9. The relative width of the 95% credible interval (CI/estimate) of abundance was low (more percision) for all simulations, except when availability and detection was low. Histograms show the distribution of relative width among 3000 simulations (500 at each combination of generating abundance and number of sites). Sampling conditions improve among panels going up (higher detection) and to the right (higher availability).

**
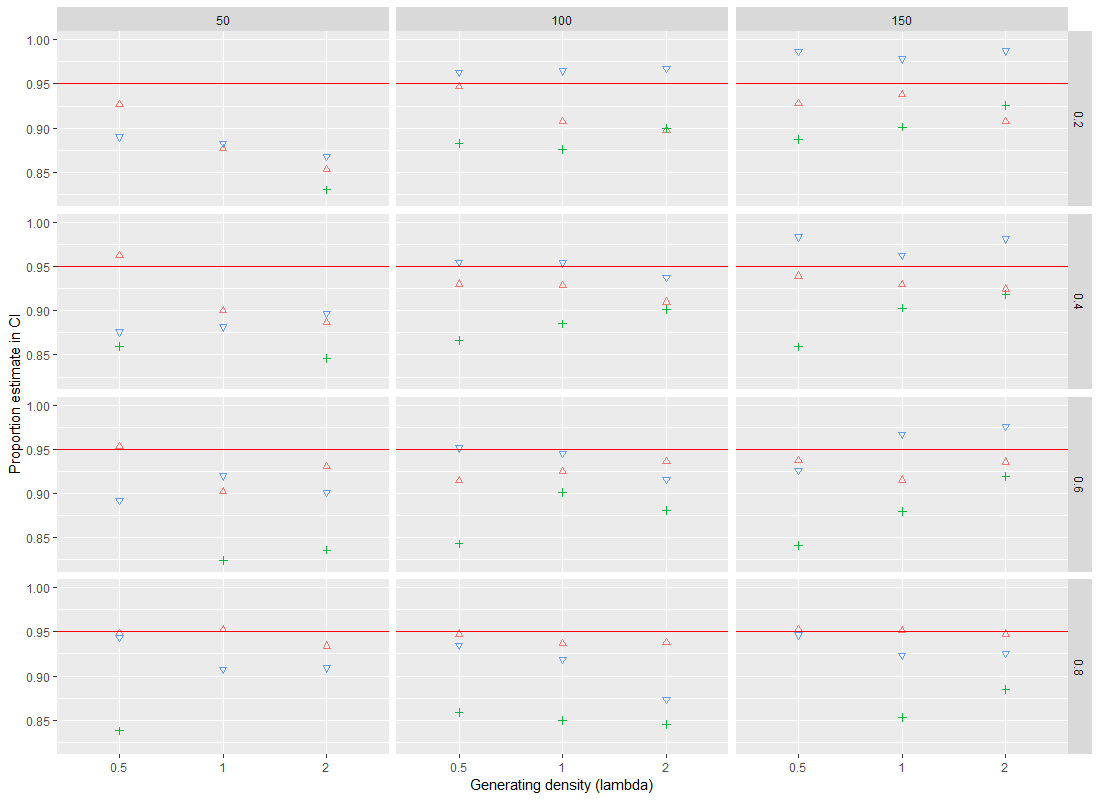
**

Figure S10. Coverage proportion for state parameters in the Bayesian model for combinations of generating density (lambda, x axis), availability (p.a, rows), and detection (sigma, columns). Each combination had 500 simulated data sets, and 25 sites. Each point gives the proportion of simulations in which 95 % credible intervals of the parameter estimate contained the generating value. Simulations in which the Bayesian model did not converge were excluded from the calculation. Red triangles show results for N, green cross is p.a, and blue triangle is sigma. The red line is at 0.95 (95 % coverage).

**
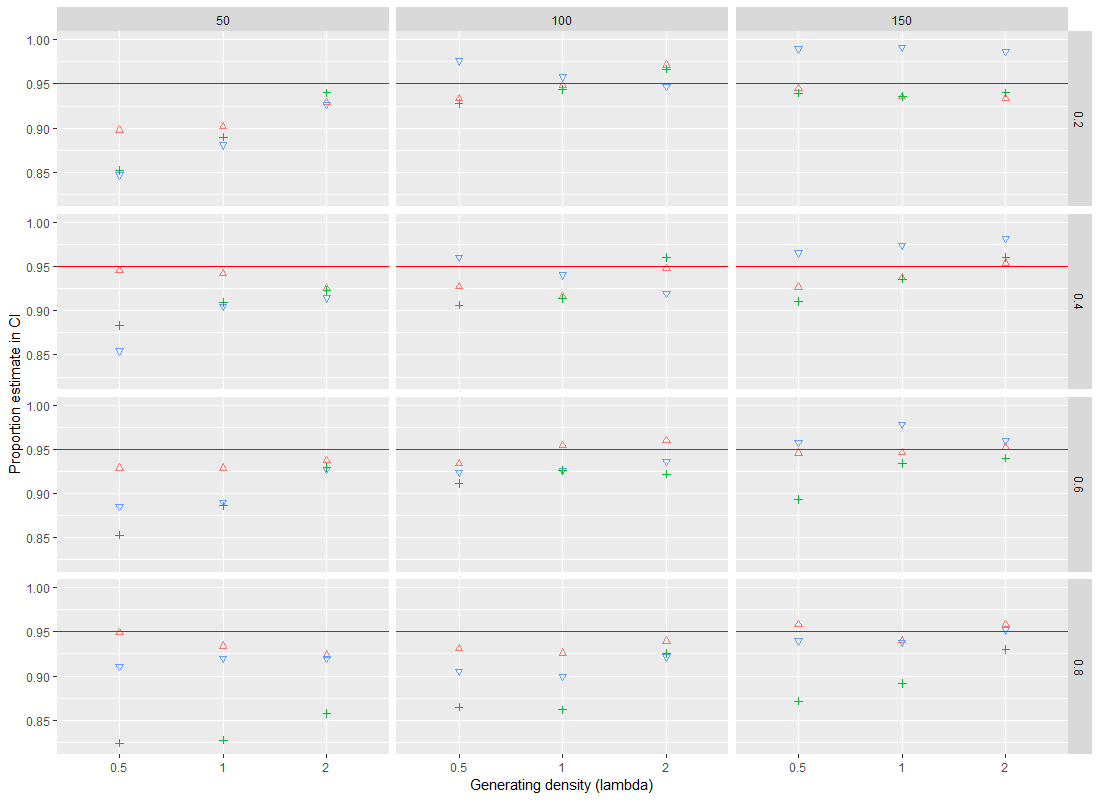
**

Figure S11. Coverage proportion for state parameters in the Bayesian model for combinations of generating density (lambda, x axis), availability (p.a, rows), and detection (sigma, columns). Each combination had 500 simulated data sets, and 50 sites. Each point gives the proportion of simulations in which 95 % credible intervals of the parameter estimate contained the generating value. Simulations in which the Bayesian model did not converge were excluded from the calculation. Red triangles show results for N, green cross is p.a, and blue triangle is sigma. The red line is at 0.95 (95 % coverage).


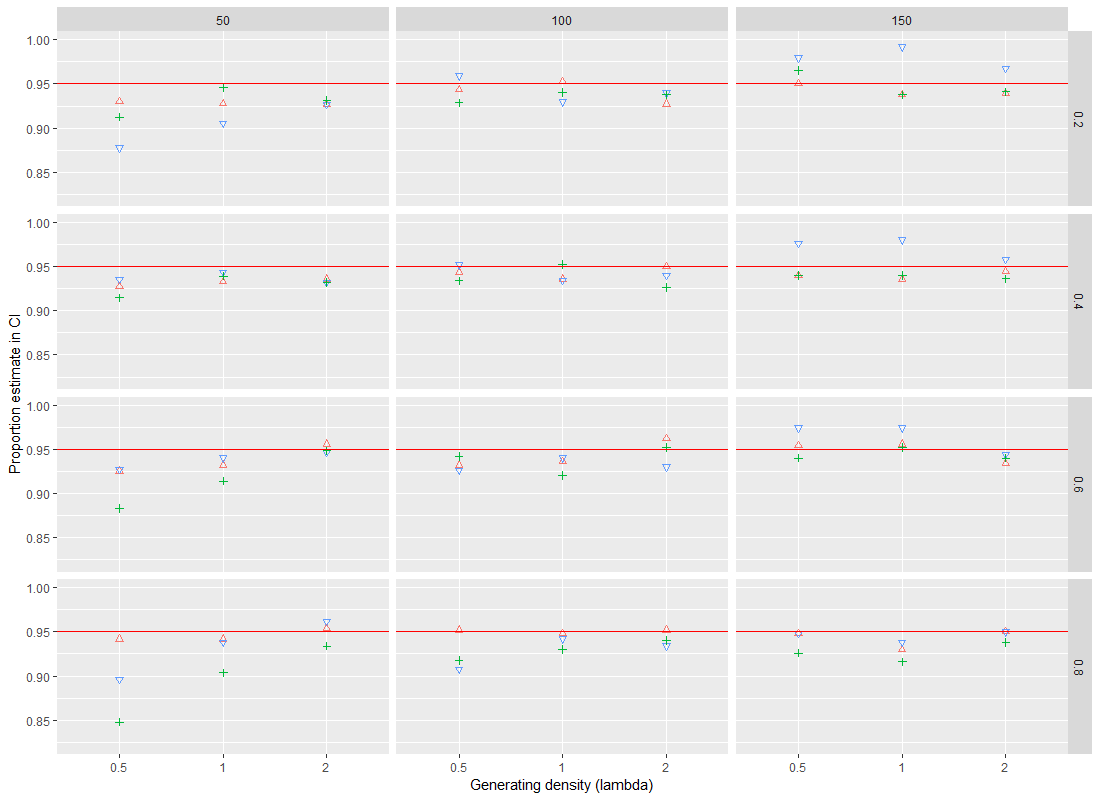


Figure S12. Coverage proportion for state parameters in the Bayesian model for combinations of generating density (lambda, x axis), availability (p.a, rows), and detection (sigma, columns). Each combination had 500 simulated data sets, and 100 sites. Each point gives the proportion of simulations in which 95 % credible intervals of the parameter estimate contained the generating value. Simulations in which the Bayesian model did not converge were excluded from the calculation. Red triangles show results for N, green cross is p.a, and blue triangle is sigma. The red line is at 0.95 (95 % coverage).


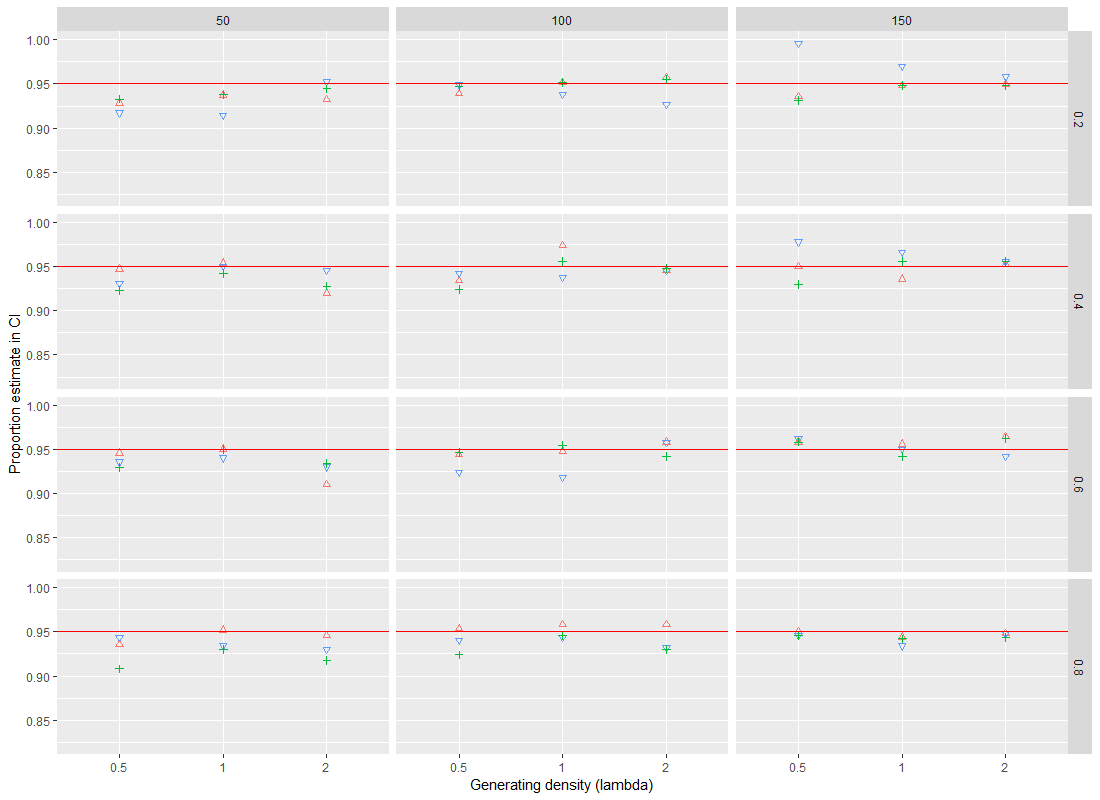


Figure S13. Coverage proportion for state parameters in the Bayesian model for combinations of generating density (lambda, x axis), availability (p.a, rows), and detection (sigma, columns). Each combination had 500 simulated data sets, and 200 sites. Each point gives the proportion of simulations in which 95 % credible intervals of the parameter estimate contained the generating value. Simulations in which the Bayesian model did not converge were excluded from the calculation. Red triangles show results for N, green cross is p.a, and blue triangle is sigma. The red line is at 0.95 (95 % coverage).


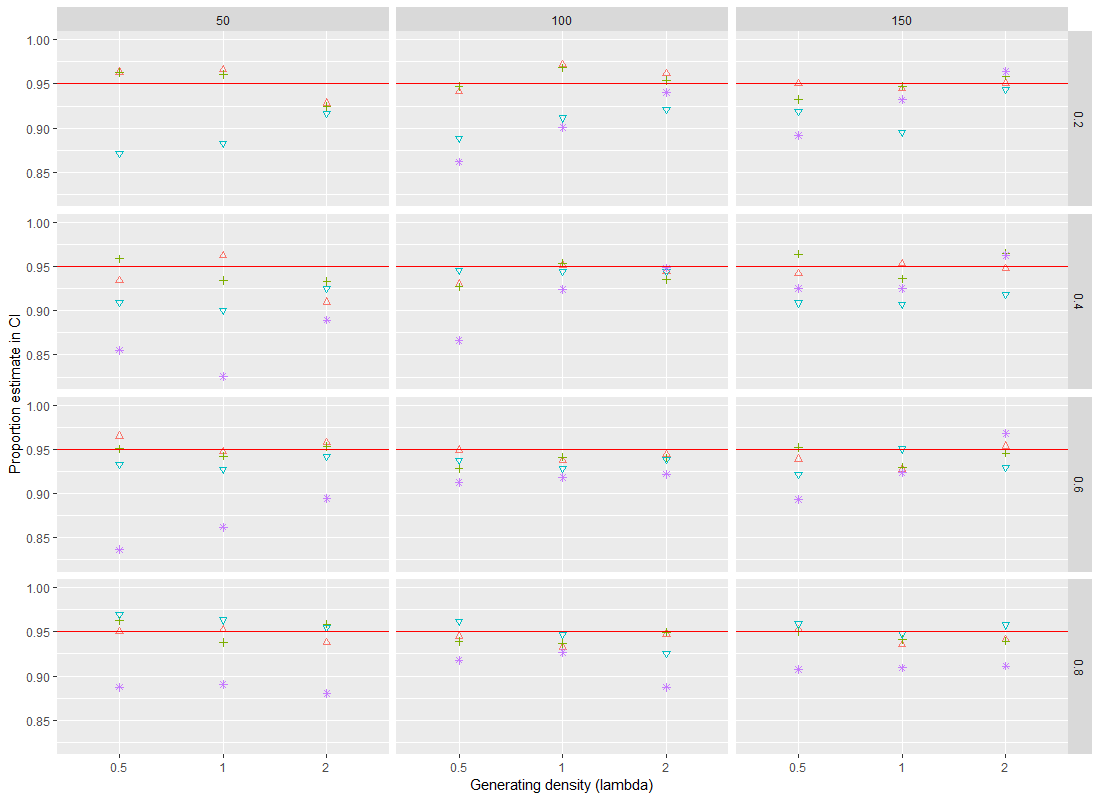


Figure S14. Coverage proportion for effect size estimates for 4 parameters in the Bayesian model for combinations of generating density (lambda, x axis), availability (p.a, rows), and detection (sigma, columns). Each combination had 500 simulated data sets, and 25 sites. Each point gives the proportion of simulations in which 95 % credible intervals of the parameter estimate contained the generating value. Simulations in which the Bayesian model did not converge were excluded from the calculation. Red triangles show results for lambda covariate 1, green cross is lambda covariate 2, and blue triangle is p.a, amd purple star is sigma. The red line is at 0.95 (95 % coverage).


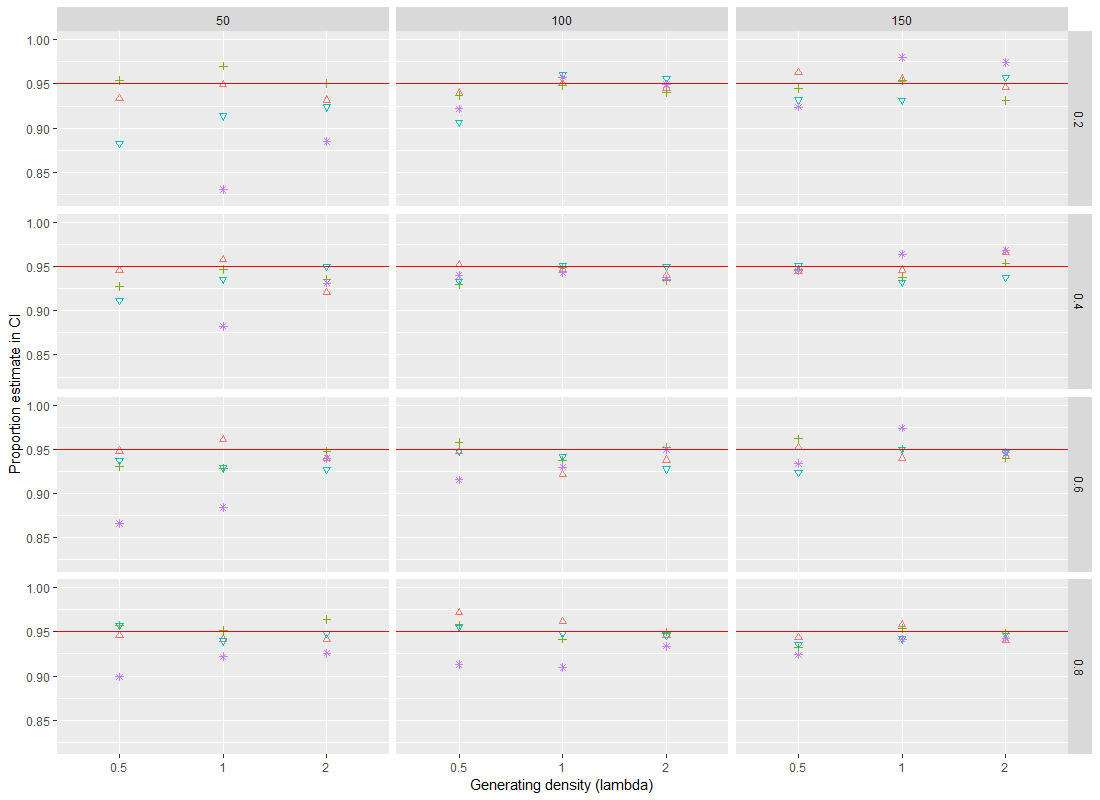


Figure S15. Coverage proportion for effect size estimates for 4 parameters in the Bayesian model for combinations of generating density (lambda, x axis), availability (p.a, rows), and detection (sigma, columns). Each combination had 500 simulated data sets, and 50 sites. Each point gives the proportion of simulations in which 95 % credible intervals of the parameter estimate contained the generating value. Simulations in which the Bayesian model did not converge were excluded from the calculation. Red triangles show results for lambda covariate 1, green cross is lambda covariate 2, and blue triangle is p.a, amd purple star is sigma. The red line is at 0.95 (95 % coverage).


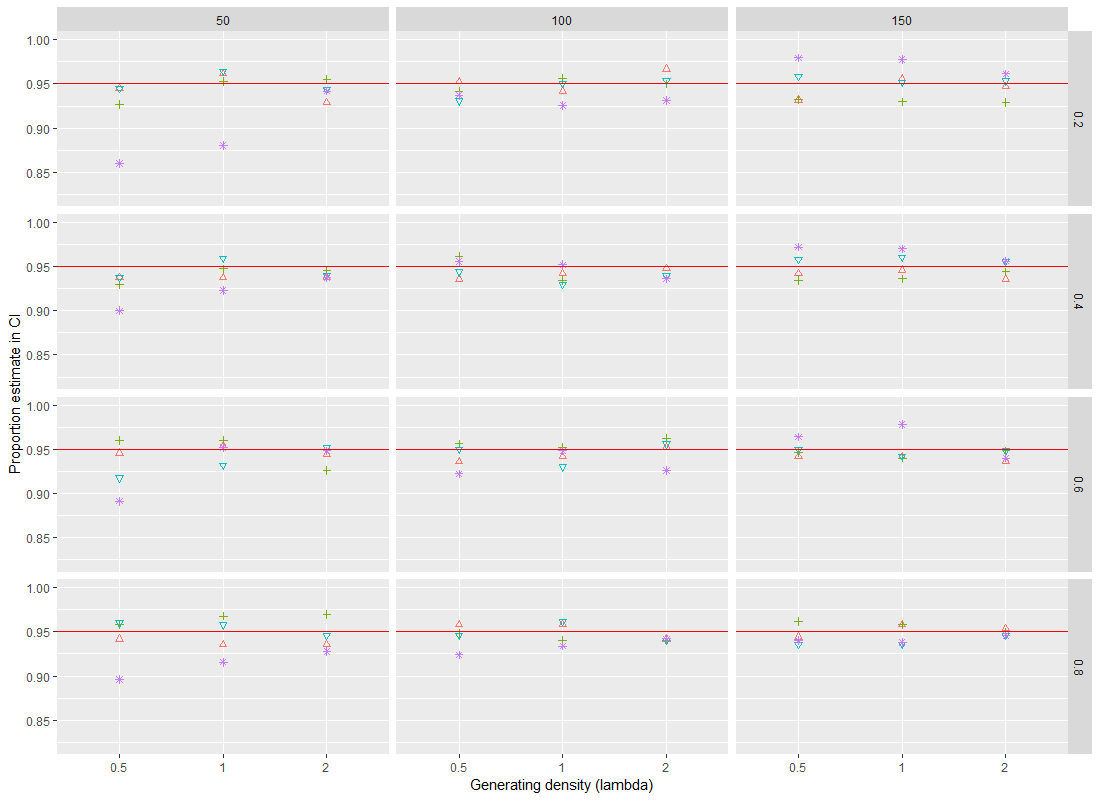


Figure S16. Coverage proportion for effect size estimates for 4 parameters in the Bayesian model for combinations of generating density (lambda, x axis), availability (p.a, rows), and detection (sigma, columns). Each combination had 500 simulated data sets, and 100 sites. Each point gives the proportion of simulations in which 95 % credible intervals of the parameter estimate contained the generating value. Simulations in which the Bayesian model did not converge were excluded from the calculation. . Red triangles show results for lambda covariate 1, green cross is lambda covariate 2, and blue triangle is p.a, amd purple star is sigma. The red line is at 0.95 (95 % coverage).


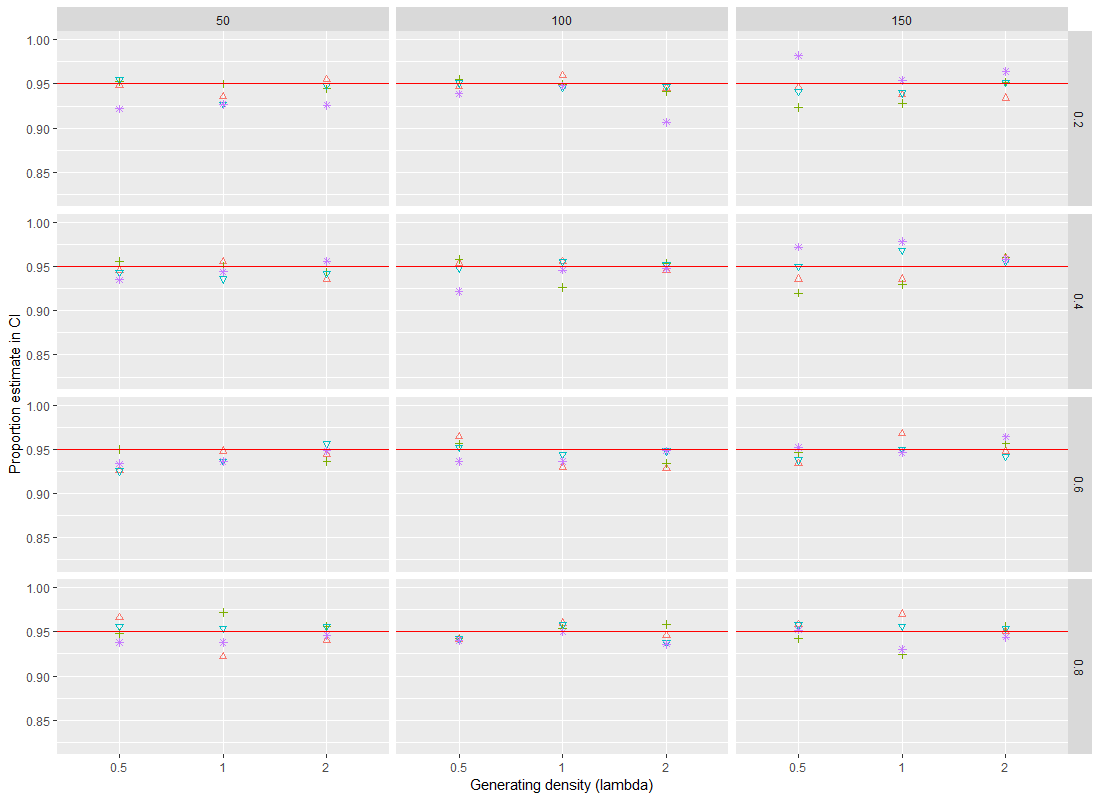


Figure S17. Coverage proportion for effect size estimates for 4 parameters in the Bayesian model for combinations of generating density (lambda, x axis), availability (p.a, rows), and detection (sigma, columns). Each combination had 500 simulated data sets, and 200 sites. Each point gives the proportion of simulations in which 95 % credible intervals of the parameter estimate contained the generating value. Simulations in which the Bayesian model did not converge were excluded from the calculation. Red triangles show results for lambda covariate 1, green cross is lambda covariate 2, and blue triangle is p.a, amd purple star is sigma. The red line is at 0.95 (95 % coverage).

**Supplemental 2. R instructions to simulate data**

simSurvey_fn <- function(Sites=100, Radius=100, Time.bins=6, Sigma=100, lambda=2, p.avail=0.7,

beta.sigma=0, beta.lambda.1=0, beta.lambda.2=0, beta.avail=0,

det.cov=rnorm(Sites,0,1) ,lambda.cov.1=rnorm(Sites,0,1), lambda.cov.2=rnorm(Sites,0,1),

avail.cov=rnorm(Sites,0,1), Plot=F){

## function to simulate data for population of mobile animals at sites with availability and detection

expit_fn <- function(x){

exp(x)/(1+exp(x))

}

logit_fn <- function(x){

log(x/(1.000000001-x))

}

det.fun <- function(distance){

exp(-distance^2/(2*mean(sigma.site)^2))

}

# loop through sites

# browser()

# populate sites

lambda.site <- exp(log(lambda) + beta.lambda.1*lambda.cov.1 + beta.lambda.2*lambda.cov.2)

# Mean.abund.site <- exp(lambda.site)

N.site <- rpois(Sites,lambda.site)

avail.site <- expit_fn(logit_fn(p.avail) + beta.avail*avail.cov)

sigma.site <- exp(log(Sigma) + beta.sigma*det.cov)

N.detected <- matrix(0, Sites, Time.bins)

distances.detected <- NULL

obs.site.id <- NULL

# loop through sites

for(i.site in seq(Sites)){

# loop through individuals

if(N.site[i.site]==0)next

for(ind in seq(N.site[i.site])){

# loop through time bins

for(t in seq(Time.bins)){

avail.now <- rbinom(1,1,avail.site[i.site])

r <- runif(1,0,1)

theta <- runif(1,0,2*pi)

coord <- cbind(sqrt(r)*cos(theta)*Radius, sqrt(r)*sin(theta)*Radius)

distance <- sqrt((0-coord[,1])^2 + (0-coord[,2])^2)

p.det <- exp(-distance^2/(2*sigma.site[i.site]^2))

detected <- rbinom(1,1,p.det*avail.now)

if(detected==1){

N.detected[i.site,t] <- N.detected[i.site,t]+1

distances.detected <- c(distances.detected,distance)

obs.site.id <- c(obs.site.id, i.site)

}

}

}

}

# browser()

cat(

"mean lambda = ", mean(lambda.site), "\n",

"mean sigma = ", mean(sigma.site), "\n",

"mean sigma BT = ", exp(mean(log(sigma.site))), "\n",

"mean detection = ", integrate(det.fun, lower=0, upper=Radius)$value/(Radius), "\n",

"mean available = ", mean(avail.site), "\n",

"N present = ", sum(N.site), "\n",

"N detected = ", sum(apply(N.detected,1,max)), "\n"

)

if(Plot==T){

op <- par(mfcol=c(1,2))

hist(distances.detected,40, main=NULL);abline(v=mean(sigma.site), col="red")

hist(N.site,20, main=NULL);abline(v=lambda, col="red")

par(op)

}

return(list(N.detected=N.detected, distances.detected=distances.detected, obs.site.id=obs.site.id,

lambda.site=lambda.site, N.site=N.site, det.cov=det.cov, lambda.cov.1=lambda.cov.1,

lambda.cov.2=lambda.cov.2, avail.cov=avail.cov))

}

test <- simSurvey_fn(lambda=5, Sigma=30, beta.sigma=-0.5, beta.lambda.1=0.5, beta.lambda.2=-0.5, beta.avail=-1, Plot=T)

**Supplemental 3. R instructions to run simulations of distance N mixture model**

**Supplemental 4. R instructions to summarize simulations**

library(tidyverse)

# Get simulation results --------------------------------------------------

Path.new <- "./RData/covariates/"

# Path.new <- "./RData/covariates/"

# Path.new.2 <- "./RData/N100_sigma_30_50_80_150/"

Dir.2 <- dir(Path.new)

# Dir.2.2 <- Dir.2(Path.new.2)

Temp.env <- new.env()

load(file=paste(Path.new,Dir.2[1],sep=""), envir=Temp.env)

Results.all.new <- Temp.env$Results

Gen.param.all.new <- Temp.env$Gen.param

converge.fail.ls.all.new <- Temp.env$converge.fail.ls

for(i1 in seq(length(Dir.2)-1)){

Temp.env <- new.env()

load(file=paste(Path.new,Dir.2[i1+1],sep=""), envir=Temp.env)

Results.all.new <- c(Results.all.new,Temp.env$Results)

Gen.param.all.new <- c(Gen.param.all.new,Temp.env$Gen.param)

converge.fail.ls.all.new <- c(converge.fail.ls.all.new,Temp.env$converge.fail.ls)

cat(paste(Path.new,Dir.2[i1+1],sep=""), "\n")

# print(ls())

}

# for(i1 in seq(length(Dir.2.2))){

# load(paste(Path.new.2,Dir.2.2[i1],sep=""))

# Results.all.new <- c(Results.all.new,Results)

# Gen.param.all.new <- c(Gen.param.all.new,Gen.param)

# converge.fail.ls.all.new <- c(converge.fail.ls.all.new,converge.fail.ls)

# # print(ls())

# }

Results <- Results.all.new

Gen.param <- Gen.param.all.new

converge.fail.ls <- converge.fail.ls.all.new

rm(Results.all.new, Gen.param.all.new, converge.fail.ls.all.new)

# Calculate bias and coverage ----------------------------------------------------------

n.iter <- length(Results)

n.iter/36

betas <- c(-1,1,-1,1) # beta.lambda.1=-1, beta.lambda.2=1, beta.avail=-1, beta.sigma=1,

Compare <- data.frame(sigma.est=numeric(n.iter), N.est=numeric(n.iter), lambda.est=numeric(n.iter),avail.est=numeric(n.iter),

p.a.gen=numeric(n.iter), lambda.gen=numeric(n.iter), sigma.gen=numeric(n.iter),

N.true=numeric(n.iter), N.obs=numeric(n.iter),

pa.in=logical(n.iter), sigma.in=logical(n.iter), lambda.in=logical(n.iter),

Converge.fail=logical(n.iter), N.in=logical(n.iter),

lambda.cov1=numeric(n.iter), lambda.cov2=numeric(n.iter),

pa.cov=numeric(n.iter), sigma.cov=numeric(n.iter),

lambda.cov1.in=logical(n.iter), lambda.cov2.in=logical(n.iter),

pa.cov.in=logical(n.iter), sigma.cov.in=logical(n.iter))

for(i in 1:n.iter){

Compare[i,"Converge.fail"] <- any(abs(Results[[i]][c(1:6,10),"Rhat"]-1)>.05)

Compare[i,1:4] <- Results[[i]][,1][c(9,14,1,13)]

Compare[i,3] <- exp(Compare[i,3])

Compare[i,5:9] <- Gen.param[[i]]

Compare[i,"pa.in"] <- ifelse(Compare[i,"p.a.gen"]!=1,

Compare[i,"p.a.gen"]>=Results[[i]][,"2.5%"]["avail.int"] & Compare[i,"p.a.gen"]<=Results[[i]][,"97.5%"]["avail.int"],

.9999>=Results[[i]][,"2.5%"]["avail.int"] & .9999<=Results[[i]][,"97.5%"]["avail.int"])

Compare[i,"sigma.in"] <- Compare[i,"sigma.gen"]>=Results[[i]][,"2.5%"]["sigma.int"] & Compare[i,"sigma.gen"]<=Results[[i]][,"97.5%"]["sigma.int"]

Compare[i,"N.in"] <- Compare[i,"N.true"]>=Results[[i]][,"2.5%"]["N.est"] & Compare[i,"N.true"]<=Results[[i]][,"97.5%"]["N.est"]

Compare[i,"lambda.in"] <- Compare[i,"lambda.gen"]>=Results[[i]][,"2.5%"]["lambda.int"] & Compare[i,"lambda.gen"]<=Results[[i]][,"97.5%"]["lambda.int"]

Compare[i,15:18] <- Results[[i]][,1][c(2,3,6,10)]

Compare[i,"lambda.cov1.in"] <- betas[1]>=Results[[i]][,"2.5%"]["beta.lambda.1"] & betas[1]<=Results[[i]][,"97.5%"]["beta.lambda.1"]

Compare[i,"lambda.cov2.in"] <- betas[2]>=Results[[i]][,"2.5%"]["beta.lambda.2"] & betas[2]<=Results[[i]][,"97.5%"]["beta.lambda.2"]

Compare[i,"pa.cov.in"] <- betas[3]>=Results[[i]][,"2.5%"]["beta.avail"] & betas[3]<=Results[[i]][,"97.5%"]["beta.avail"]

Compare[i,"sigma.cov.in"] <- betas[4]>=Results[[i]][,"2.5%"]["beta.sigma"] & betas[4]<=Results[[i]][,"97.5%"]["beta.sigma"]

}

Compare <- group_by(Compare, p.a.gen, lambda.gen, sigma.gen) %>%

mutate(., iter.seq = 1:n()) %>%

filter(., iter.seq < 501) %>%

as.data.frame(.)

xtabs(~p.a.gen + lambda.gen + sigma.gen, Compare)

Compare.conv <- filter(Compare, Converge.fail==F)

xtabs(~p.a.gen + lambda.gen + sigma.gen, Compare.conv)

apply(Compare,2,mean)

apply(Compare.conv,2,mean)

Compare.conv <- mutate(Compare.conv,

p.a.abs.error = (avail.est-p.a.gen),

p.a.rel.error = (avail.est-p.a.gen)/p.a.gen,

p.a.sqr.error = (avail.est-p.a.gen)^2,

lambda.abs.error = (lambda.est-lambda.gen),

lambda.rel.error = (lambda.est-lambda.gen)/lambda.gen,

lambda.sqr.error = (lambda.est-lambda.gen)^2,

sigma.abs.error = (sigma.est-sigma.gen),

sigma.rel.error = (sigma.est-sigma.gen)/sigma.gen,

sigma.sqr.error = (sigma.est-sigma.gen)^2,

N.abs.error = (N.est-N.true),

N.rel.error = (N.est-N.true)/N.true,

N.sqr.error = (N.est-N.true)^2,

lambda.cov1.abs.error = (lambda.cov1-betas[1]),

lambda.cov1.rel.error = (lambda.cov1-betas[1])/betas[1],

lambda.cov1.sqr.error = (lambda.cov1-betas[1])^2,

lambda.cov2.abs.error = (lambda.cov2-betas[2]),

lambda.cov2.rel.error = (lambda.cov2-betas[2])/betas[2],

lambda.cov2.sqr.error = (lambda.cov2-betas[2])^2,

pa.cov.abs.error = (pa.cov-betas[3]),

pa.cov.rel.error = (pa.cov-betas[3])/betas[3],

pa.cov.sqr.error = (pa.cov-betas[3])^2,

sigma.cov.abs.error = (sigma.cov-betas[4]),

sigma.cov.rel.error = (sigma.cov-betas[4])/betas[4],

sigma.cov.sqr.error = (sigma.cov-betas[4])^2

)

compare.summary <- group_by(Compare.conv, p.a.gen, lambda.gen, sigma.gen) %>%

summarize(., N=n(), N.mean=mean(N.est), N.in.summ=mean(N.in),

lambda.mean=mean(lambda.est), lambda.in.summ=mean(lambda.in),

sigma.mean=mean(sigma.est), sigma.in.summ=mean(sigma.in),

pa.mean=mean(avail.est), pa.in.summ=mean(pa.in),

lambda.cov1.mean=mean(lambda.cov1), lambda.cov1.in.summ=mean(lambda.cov1.in),

lambda.cov2.mean=mean(lambda.cov2), lambda.cov2.in.summ=mean(lambda.cov2.in),

pa.cov.mean=mean(pa.cov), pa.cov.in.summ=mean(pa.cov.in),

sigma.cov.mean=mean(sigma.cov), sigma.cov.in.summ=mean(sigma.cov.in),

lambda.MAE = mean(lambda.abs.error),

lambda.MRE = mean(lambda.rel.error),

lambda.MSE = mean(lambda.sqr.error),

lambda.RMSE = sqrt(lambda.MSE),

lambda.var = var(lambda.est),

lambda.sd = sd(lambda.est),

sigma.MAE = mean(sigma.abs.error),

sigma.MRE = mean(sigma.rel.error),

sigma.MSE = mean(sigma.sqr.error),

sigma.RMSE = sqrt(sigma.MSE),

sigma.var = var(sigma.est),

sigma.sd = sd(sigma.est),

p.a.MAE = mean(p.a.abs.error),

p.a.MRE = mean(p.a.rel.error),

p.a.MSE = mean(p.a.sqr.error),

p.a.RMSE = sqrt(p.a.MSE),

p.a.var = var(avail.est),

p.a.sd = sd(avail.est),

N.MAE = mean(N.abs.error),

N.MRE = mean(N.rel.error),

N.MSE = mean(N.sqr.error),

N.RMSE = sqrt(N.MSE),

N.var = var(N.est),

N.sd = sd(N.est),

lambda.cov1.MAE =mean(lambda.cov1.abs.error),

lambda.cov1.MRE =mean(lambda.cov1.rel.error),

lambda.cov1.MSE =mean(lambda.cov1.sqr.error),

lambda.cov1.RMSE = sqrt(lambda.cov1.MSE),

lambda.cov1.var = var(lambda.cov1),

lambda.cov1.sd = sd(lambda.cov1),

lambda.cov2.MAE =mean(lambda.cov2.abs.error),

lambda.cov2.MRE =mean(lambda.cov2.rel.error),

lambda.cov2.MSE =mean(lambda.cov2.sqr.error),

lambda.cov2.RMSE = sqrt(lambda.cov2.MSE),

lambda.cov2.var = var(lambda.cov2),

lambda.cov2.sd = sd(lambda.cov2),

pa.cov.MAE = mean(pa.cov.abs.error),

pa.cov.MRE = mean(pa.cov.rel.error),

pa.cov.MSE = mean(pa.cov.sqr.error),

pa.cov.RMSE = sqrt(pa.cov.MSE),

pa.cov.var = var(pa.cov),

pa.cov.sd = sd(pa.cov),

sigma.cov.MAE = mean(sigma.cov.abs.error),

sigma.cov.MRE = mean(sigma.cov.rel.error),

sigma.cov.MSE = mean(sigma.cov.sqr.error),

sigma.cov.RMSE = sqrt(sigma.cov.MSE),

sigma.cov.var = var(sigma.cov),

sigma.cov.sd = sd(sigma.cov)

)

write.csv(compare.summary, file="./output/second paper/sims/bias_stats_100_covariates.csv")

# convergence failure -----------------------------------------------------

Compare.noconv <- filter(Compare, Converge.fail==T)

xtabs(~sigma.gen+p.a.gen, Compare.noconv)

not.converged <- compare.summary[which(compare.summary$N != 100),1:4]

not.converged$failed <- 500 - not.converged$N

ppi=100

tiff("./Output/second paper/sims/convergence failure 100 sites lowhres.tiff", width = 11*ppi, height = 8*ppi, res = ppi)

ggplot(not.converged, aes(x=as.factor(lambda.gen), y=failed)) + geom_point() +

facet_grid(as.factor(p.a.gen)~as.factor(sigma.gen)) +

theme(legend.position="none") +

xlab("Generating lambda") + ylab("Number failed out of 100")

dev.off()

write.csv(not.converged, file="./Output/second paper/sims/convergence failure 100 covariates.csv")

# plot coverage ------------------------------------------------------------

## explore coverage

ggplot(compare.summary, aes(x=as.factor(lambda.gen), y=N.in.summ)) + geom_point() +

geom_hline(yintercept = 0.95, colour="red") + facet_grid(as.factor(p.a.gen)~as.factor(sigma.gen)) +

theme(legend.position="none") +

xlab("Generating lambda") + ylab("Proportion N estimate in CI") +

ggtitle("distance N mixture model simulations") + ylim(0.8,1)

ggplot(compare.summary, aes(x=as.factor(lambda.gen), y=pa.in.summ)) + geom_point() +

geom_hline(yintercept = 0.95, colour="red") + facet_grid(as.factor(p.a.gen)~as.factor(sigma.gen)) +

theme(legend.position="none") +

xlab("Generating lambda") + ylab("Proportion p.a estimate in CI") +

ggtitle("distance N mixture model simulations")

ggplot(compare.summary, aes(x=as.factor(lambda.gen), y=sigma.in.summ)) + geom_point() +

geom_hline(yintercept = 0.95, colour="red") + facet_grid(as.factor(p.a.gen)~as.factor(sigma.gen)) +

theme(legend.position="none") +

xlab("Generating lambda") + ylab("Proportion sigma estimate in CI") +

ggtitle("distance N mixture model simulations")

names(compare.summary) # , 14, 16, 18, 20

compare.summary.long <- pivot_longer(compare.summary, cols = c(6, 10, 12), names_to = "parameter", values_to = "coverage")

ggplot(compare.summary.long, aes(x=as.factor(lambda.gen), y=coverage, col=parameter)) +

geom_point(aes(shape=parameter)) + scale_shape_manual(values=c(2,3,6))+

geom_hline(yintercept = 0.95, colour="red") + facet_grid(as.factor(p.a.gen)~as.factor(sigma.gen)) +

theme(legend.position="bottom") +

xlab("Generating lambda") + ylab("Proportion estimate in CI") +

ggtitle("distance N mixture model simulations")

ppi=100

tiff("./Output/second paper/sims/coverage parameters 100 sites lowres.tiff", width = 11*ppi, height = 8*ppi, res = ppi)

ggplot(compare.summary.long, aes(x=as.factor(lambda.gen), y=coverage, col=parameter)) +

geom_point(aes(shape=parameter)) + scale_shape_manual(values=c(2,3,6))+

geom_hline(yintercept = 0.95, colour="red", size=0.25) + facet_grid(as.factor(p.a.gen)~as.factor(sigma.gen)) +

theme(legend.position="none") + ylim(0.82,1)+

xlab("Generating density (lambda)") + ylab("Proportion estimate in CI")

dev.off()

sink("./Output/second paper/sims/coverage legend parameters.txt")

cat(

"red triangle is N, greem plus is pa, blue triangle is sigma, red line at 0.9",

"\n",

"lowres = 100 ppi, medres = 300 ppi, highres=600 ppi"

)

sink()

names(compare.summary) # , 14, 16, 18, 20

compare.summary.long.2 <- pivot_longer(compare.summary, cols = c(14, 16, 18, 20), names_to = "parameter", values_to = "coverage")

ggplot(compare.summary.long.2, aes(x=as.factor(lambda.gen), y=coverage, col=parameter)) +

geom_point(aes(shape=parameter)) + scale_shape_manual(values=c(2,3,6,8))+

geom_hline(yintercept = 0.95, colour="red") + facet_grid(as.factor(p.a.gen)~as.factor(sigma.gen)) +

theme(legend.position="bottom") +

xlab("Generating lambda") + ylab("Proportion estimate in CI") +

ggtitle("distance N mixture model simulations")

ppi=100

tiff("./Output/second paper/sims/coverage covariates 100 sites lowres.tiff", width = 11*ppi, height = 8*ppi, res = ppi)

ggplot(compare.summary.long.2, aes(x=as.factor(lambda.gen), y=coverage, col=parameter)) +

geom_point(aes(shape=parameter)) + scale_shape_manual(values=c(2,3,6,8))+

geom_hline(yintercept = 0.95, colour="red", size=0.25) + facet_grid(as.factor(p.a.gen)~as.factor(sigma.gen)) +

theme(legend.position="none") + ylim(0.82,1)+

xlab("Generating density (lambda)") + ylab("Proportion estimate in CI")

dev.off()

sink("./Output/second paper/sims/coverage legend covariates.txt")

cat(

"red triangle is lambda covariate 1, greem plus is lambda covariate 2, blue triangle is pa covariate, purple star is sigma covariate

red line at 0.9",

"\n",

"lowres = 100 ppi, medres = 300 ppi, highres=600 ppi"

)

sink()

# explore bias ------------------------------------------------------------

N=100

lambda=c(0.5,1,2)

expected.N <- apply(round(exp(matrix(log(lambda), nrow=N, ncol=3, byrow = T) + rnorm(N, 0,1) - rnorm(N,0,1))),2,sum)

mean.simmulated.N <- group_by(Compare, lambda.gen) %>%

summarize(., N=n(), mean=mean(N.true)) %>%

as.data.frame(.)

ppi=600

tiff("./Output/second paper/sims/abundance plot 100 sites.tiff", width = 11*ppi, height = 8*ppi, res = ppi)

ggplot(Compare.conv, aes(x=as.factor(lambda.gen), y=N.est)) + geom_boxplot() +

geom_point(aes(1, mean.simmulated.N[1,3], colour="red")) + geom_point(aes(2, mean.simmulated.N[2,3], colour="red")) +

geom_point(aes(3, mean.simmulated.N[3,3], colour="red")) +

facet_grid(as.factor(p.a.gen)~as.factor(sigma.gen)) + theme(legend.position="none") +

xlab("Generating lambda") + ylab("Estimated abundance") + ylim(c(0,2000))

dev.off()

ppi=600

tiff("./Output/second paper/sims/abundance plot 100 sites square highres.tiff", width = 6*ppi, height = 6*ppi, res = ppi)

ggplot(Compare.conv, aes(x=as.factor(lambda.gen), y=N.est)) + geom_boxplot() +

geom_point(aes(1, mean.simmulated.N[1,3], colour="red")) + geom_point(aes(2, mean.simmulated.N[2,3], colour="red")) +

geom_point(aes(3, mean.simmulated.N[3,3], colour="red")) +

facet_grid(as.factor(p.a.gen)~as.factor(sigma.gen)) + theme(legend.position="none") +

xlab("Generating lambda") + ylab("Estimated abundance") + ylim(c(0,2000))

dev.off()

ggplot(Compare.conv, aes(x=N.est)) + geom_histogram(binwidth = 50) +

# geom_point(aes(1, mean.simmulated.N[1,3], colour="red")) + geom_point(aes(2, mean.simmulated.N[2,3], colour="red")) +

# geom_point(aes(3, mean.simmulated.N[3,3], colour="red")) +

facet_grid(as.factor(p.a.gen)~as.factor(sigma.gen):as.factor(lambda.gen)) + theme(legend.position="none") +

xlab("Generating lambda") + ylab("Estimated abundance")

ggplot(Compare.conv, aes(x=as.factor(lambda.gen), y=N.est)) +

geom_violin(draw_quantiles = c(0.25, 0.5, 0.75), adjust=1.25) +

geom_point(aes(1, mean.simmulated.N[1,3], colour="red")) + geom_point(aes(2, mean.simmulated.N[2,3], colour="red")) +

geom_point(aes(3, mean.simmulated.N[3,3], colour="red")) +

facet_grid(as.factor(p.a.gen)~as.factor(sigma.gen)) + theme(legend.position="none") +

xlab("Generating lambda") + ylab("Estimated abundance") + ylim(c(0,2000))

# plot relative bias ------------------------------------------------------

names(Compare.conv)[grep("rel.error",names(Compare.conv))]

grep("rel.error",names(Compare.conv))

bias.long <- pivot_longer(Compare.conv, cols = c(25, 28, 31, 34), names_to = "parameter", values_to = "MRE")

# bias.long.all <- pivot_lnger(Compare.conv, cols = c(17, 20, 23, 26), names_to = "parameter", values_to = "MRE")

ggplot(bias.long, aes(x=as.factor(lambda.gen), y=MRE, col=parameter)) + geom_boxplot() +

facet_grid(as.factor(p.a.gen)~as.factor(sigma.gen)) + theme(legend.position="bottom") +

xlab("Generating lambda") + ylab("Relative bias") +

geom_hline(yintercept = 0, colour="red", size=0.25)

ppi=100

tiff("./Output/second paper/sims/relative bias 100 parameters lowres.tiff", width = 11*ppi, height = 8*ppi, res = ppi)

ggplot(bias.long, aes(x=as.factor(lambda.gen), y=MRE, col=parameter)) + geom_boxplot() +

facet_grid(as.factor(p.a.gen)~as.factor(sigma.gen)) + theme(legend.position="none") +

xlab("Generating lambda") + ylab("Relative bias") +

geom_hline(yintercept = 0, colour="red", size=0.25)

dev.off()

sink("./Output/second paper/sims/relative bias parameters.txt")

cat(

"order is lamdda, N, pa, sigma, red line at 0",

"\n",

"lowres = 100 ppi, medres = 300 ppi, highres=600 ppi"

)

sink()

names(Compare.conv)[grep("rel.error",names(Compare.conv))]

grep("rel.error",names(Compare.conv))

bias.long.2 <- filter(Compare.conv, sigma.gen>30) %>%

pivot_longer(., cols = c(37, 40, 43, 46), names_to = "parameter", values_to = "MRE")

bias.long.2.all <- pivot_longer(Compare.conv, cols = c(17, 20, 23, 26), names_to = "parameter", values_to = "MRE")

ggplot(bias.long.2, aes(x=as.factor(lambda.gen), y=MRE, col=parameter)) + geom_boxplot() +

facet_grid(as.factor(p.a.gen)~as.factor(sigma.gen)) + theme(legend.position="bottom") +

xlab("Generating lambda") + ylab("Relative bias") +

geom_hline(yintercept = 0, colour="red", size=0.25)

ppi=100

tiff("./Output/second paper/sims/relative bias 100 covariates lowres.tiff", width = 11*ppi, height = 8*ppi, res = ppi)

ggplot(bias.long.2, aes(x=as.factor(lambda.gen), y=MRE, col=parameter)) + geom_boxplot() +

facet_grid(as.factor(p.a.gen)~as.factor(sigma.gen)) + theme(legend.position="none") +

xlab("Generating lambda") + ylab("Relative bias") +

geom_hline(yintercept = 0, colour="red", size=0.25)

dev.off()

sink("./Output/second paper/sims/relative bias parameters.txt")

cat(

"order is lamdda, N, pa, sigma, red line at 0",

"\n",

"lowres = 100 ppi, medres = 300 ppi, highres=600 ppi"

)

sink()

N=100

lambda=c(0.5,1,2)

expected.N <- apply(round(exp(matrix(log(lambda), nrow=N, ncol=3, byrow = T) + rnorm(N, 0,1) - rnorm(N,0,1))),2,sum)

mean.simmulated.N <- group_by(Compare, lambda.gen) %>%

summarize(., N=n(), mean=mean(N.true)) %>%

as.data.frame(.)

ppi=100

tiff("./Output/abundance plot 200 sites.tiff", width = 11*ppi, height = 8*ppi, res = ppi)

ggplot(Compare.conv, aes(x=as.factor(lambda.gen), y=N.est)) + geom_boxplot() +

geom_point(aes(1, mean.simmulated.N[1,3], colour="red")) + geom_point(aes(2, mean.simmulated.N[2,3], colour="red")) +

geom_point(aes(3, mean.simmulated.N[3,3], colour="red")) +

facet_grid(as.factor(p.a.gen)~as.factor(sigma.gen)) + theme(legend.position="none") +

xlab("Generating lambda") + ylab("Estimated abundance") + ylim(c(0,2000))

dev.off()

**Supplemental 5. R instructions to conduct model selection for**

**terrapin survey data**

# full BUGS model --------------------------------------------------------------

sink("./BUGS/terrapin__final_OD_surveyRE.txt")

cat("

model {

## PRIORS

# priors for distance function

# sigma.int ~ dunif(0.48,5.7)

sigma.int ~ dunif(1.6,300) # intercept for sigma in Distance function (Sigma cannot be <1.6 or det=0 for some diatnaces)

# priors for availability covariate model

beta.a0 ~ dbeta(1,1)

beta.a0.logit <- logit(beta.a0)

survey.sd ~ dnorm(0, 0.2)I(0,) # hyperprior for RE sd

survey.tau <- 1 / (survey.sd * survey.sd)

for(p in 1:n.surveys){

survey.re[p] ~ dnorm(0,survey.tau)

}

OD.sd ~ dnorm(0, 0.2)I(0,) # hyperprior for OD RE sd

OD.tau <- 1 / (OD.sd * OD.sd)

for(p in 1:n.surveys){

for(t in 1:n.rep.counts){

OD.re[p,t] ~ dnorm(0,OD.tau)

}

}

beta.int ~ dt(0,1/(2.5^2),7)

beta.all_land ~ dt(0,1/(2.5^2),7)

beta.mangrove ~ dt(0,1/(2.5^2),7)

beta.seagrass2009 ~ dt(0,1/(2.5^2),7)

beta.seagrass2015 ~ dt(0,1/(2.5^2),7)

beta.D.occupied ~ dt(0,1/(2.5^2),7)

## Detection functions (Detection = availability + perceptibility)

for(k in 1:n.surveys){

# covariates for scale parameter of Distance function (perceptibility)

log(sigma[k]) <- (sigma.int)

# covariates for availability parameters of Tme-Removal (availability)

for(t in 1:n.rep.counts){

logit(pavail[k,t]) <- beta.a0.logit + OD.re[k,t]

}

## Distance sampling probability estimation using summation technique: Pr(p of x)=exp(-x^2/2*sigma^2)*f(x)

for(b in 1:n.breaks){

log(g[b,k])<- -mdpts[b]*mdpts[b]/(2*sigma[k]*sigma[k]) # half-normal detection function - first half of eq.,

f[b,k]<- ( 2*mdpts[b]*delta )/(maxd*maxd) # this is f(x), the scaled radial density function (distribution of area around point)

# need [b] index on delta if distance intervals are not all of equal size

pi.pd[b,k]<- g[b,k]*f[b,k] #this is the product Pr(detect)*Pr(distribution)

pi.pd.c[b,k]<- pi.pd[b,k]/pdet[k] # standardizing based on overall capture probability - conditional formulation

}

pdet[k]<-sum(pi.pd[,k]) # probability of detection is the sum of all the rectangular areas calculated above

}

### model likelihood

## Observation-level model

for(i in 1:n.obs){

dclass[i] ~ dcat(pi.pd.c[,obs.surveyid[i]])

}

## Abundance estimation

for(k in 1:n.surveys){

# site abundance model

log(lambda[k])<- beta.int + beta.mangrove*mangrove[k] + beta.all_land*all_land[k] +

beta.seagrass2009*seagrass2009[k]*(1-seagrass2015[k]) + beta.seagrass2015*seagrass2015[k] +

beta.D.occupied*D.occupied[k] + survey.re[k]

N[k]~dpois(lambda[k])# predicted abundance per point

# loop through surveys

for(j in 1:n.rep.counts){

# binomial model for # of captured individuals

y[k,j]~ dbin(pdet[k]*pavail[k,j],N[k])

y.new[k,j] ~ dbin(pdet[k]*pavail[k,j],N[k])

y.expected[k,j] <- pdet[k]*pavail[k,j]*N[k]

TF.stat[k,j] <- pow(sqrt(y[k,j]) - sqrt( y.expected[k,j]),2)

TF.stat.new[k,j] <- pow(sqrt(y.new[k,j]) - sqrt(y.expected[k,j]),2)

}

}

## posterior predictive check (Bayesian p value, BPV)

fit.obs<- sum(TF.stat[,])

fit.new<- sum(TF.stat.new[,])

BPV <- (fit.obs < fit.new)

}", fill=TRUE)

sink()

# MS BUGS model --------------------------------------------------------------

sink("./BUGS/Observed_terrapin__MS.txt")

cat("

model {

## PRIORS

# priors for distance function

sigma.int ~ dunif(1.6,300)

# sigma.int ~ dunif(0.48,5.7) # intercept for sigma in Distance function (Sigma cannot be <1.6 or det=0 for some diatnaces)

# priors for availability covariate model

beta.a0 ~ dbeta(1,1)

beta.a0.logit <- logit(beta.a0)

survey.sd ~ dnorm(0, 0.2)I(0,) # hyperprior for RE sd

survey.tau <- 1 / (survey.sd * survey.sd)

for(p in 1:n.surveys){

survey.re[p] ~ dnorm(0,survey.tau)

}

OD.sd ~ dnorm(0, 0.2)I(0,) # hyperprior for OD RE sd

OD.tau <- 1 / (OD.sd * OD.sd)

for(p in 1:n.surveys){

for(t in 1:n.rep.counts){

OD.re[p,t] ~ dnorm(0,OD.tau)

}

}

beta.int ~ dt(0,1/(2.5^2),7)

beta.all_land ~ dnorm(mu.all.land[ms.all_land+1],tau.all.land[ms.all_land+1])

beta.mangrove ~ dnorm(mu.mangrove [ms.mangrove+1],tau.mangrove [ms.mangrove+1])

beta.seagrass2009 ~ dnorm(mu.seagrass2009[ms.seagrass+1],tau.seagrass2009[ms.seagrass+1])

beta.seagrass2015 ~ dnorm(mu.seagrass2015[ms.seagrass+1],tau.seagrass2015[ms.seagrass+1])

beta.D.occupied ~ dnorm(mu.D.occupied[ms.D.occupied+1],tau.D.occupied[ms.D.occupied+1])

# model selection dummy variables

ms.all_land ~ dbern(0.5)

ms.mangrove ~ dbern(0.5)

ms.seagrass ~ dbern(0.5)

ms.D.occupied ~ dbern(0.5)

### Detection functions (Detection = availability + perceptibility)

for(k in 1:n.surveys){

# covariates for scale parameter of Distance function (perceptibility)

# log(sigma[k]) <- (sigma.int)

sigma[k] <- sigma.int

# covariates for availability parameters of Tme-Removal (availability)

for(t in 1:n.rep.counts){

logit(pavail[k,t]) <- beta.a0.logit + OD.re[k,t]

}

## Distance sampling probability estimation using summation technique: Pr(p of x)=exp(-x^2/2*sigma^2)*f(x)

for(b in 1:n.breaks){

log(g[b,k])<- -mdpts[b]*mdpts[b]/(2*sigma[k]*sigma[k]) # half-normal detection function - first half of eq.,

f[b,k]<- ( 2*mdpts[b]*delta )/(maxd*maxd) # this is f(x), the scaled radial density function (distribution of area around point)

# need [b] index on delta if distance intervals are not all of equal size

pi.pd[b,k]<- g[b,k]*f[b,k] #this is the product Pr(detect)*Pr(distribution)

pi.pd.c[b,k]<- pi.pd[b,k]/pdet[k] # standardizing based on overall capture probability - conditional formulation

}

pdet[k]<-sum(pi.pd[,k]) # probability of detection is the sum of all the rectangular areas calculated above

}

### model likelihood

## Observation-level model

for(i in 1:n.obs){

#single binomial trial with categorical distribution linking distance class and time interval to survey point

dclass[i] ~ dcat(pi.pd.c[,obs.surveyid[i]])

}

## Abundance estimation

for(k in 1:n.surveys){

# site abundance model

log(lambda[k])<- beta.int + ms.mangrove*beta.mangrove*mangrove[k] + ms.all_land*beta.all_land*all_land[k] +

ms.seagrass*beta.seagrass2009*seagrass2009[k]*(1-seagrass2015[k]) + ms.seagrass*beta.seagrass2015*seagrass2015[k] +

beta.D.occupied*D.occupied[k] + survey.re[k]

N[k]~dpois(lambda[k])# predicted abundance per point

# loop through surveys

for(j in 1:n.rep.counts){

# binomial model for # of captured individuals

y[k,j]~ dbin(pdet[k]*pavail[k,j],N[k]) # counts related to probability of detection, given availability

y.new[k,j] ~ dbin(pdet[k]*pavail[k,j],N[k])

y.expected[k,j] <- pdet[k]*pavail[k,j]*N[k]

TF.stat[k,j] <- pow(sqrt(y[k,j]) - sqrt( y.expected[k,j]),2)

TF.stat.new[k,j] <- pow(sqrt(y.new[k,j]) - sqrt(y.expected[k,j]),2)

}

}

## posterior predictive check (Bayesian p value, BPV)

fit.obs<- sum(TF.stat[,])

fit.new<- sum(TF.stat.new[,])

BPV <- (fit.obs < fit.new)

}", fill=TRUE)

sink()

# check betas BUGS model --------------------------------------------------------------

sink("./BUGS/Observed_terrapin__betas.txt")

cat("

model {

## PRIORS

# priors for distance function

sigma.int ~ dunif(1.6,300)

# sigma.int ~ dunif(0.48,5.7) # intercept for sigma in Distance function (Sigma cannot be <1.6 or det=0 for some diatnaces)

# priors for availability covariate model

beta.a0 ~ dbeta(1,1)

beta.a0.logit <- logit(beta.a0)

survey.sd ~ dnorm(0, 0.2)I(0,) # hyperprior for RE sd

survey.tau <- 1 / (survey.sd * survey.sd)

for(p in 1:n.surveys){

survey.re[p] ~ dnorm(0,survey.tau)

}

OD.sd ~ dnorm(0, 0.2)I(0,) # hyperprior for OD RE sd

OD.tau <- 1 / (OD.sd * OD.sd)

for(p in 1:n.surveys){

for(t in 1:n.rep.counts){

OD.re[p,t] ~ dnorm(0,OD.tau)

}

}

beta.int ~ dt(0,1/(2.5^2),7)

beta.all_land ~ dt(0,1/(2.5^2),7)

beta.mangrove ~ dt(0,1/(2.5^2),7)

beta.seagrass2009 ~ dt(0,1/(2.5^2),7)

beta.seagrass2015 ~ dt(0,1/(2.5^2),7)

beta.D.occupied ~ dt(0,1/(2.5^2),7)

### Detection functions (Detection = availability + perceptibility)

for(k in 1:n.surveys){

# covariates for scale parameter of Distance function (perceptibility)

# log(sigma[k]) <- (sigma.int)

sigma[k] <- sigma.int

# covariates for availability parameters of Tme-Removal (availability)

for(t in 1:n.rep.counts){

logit(pavail[k,t]) <- beta.a0.logit + OD.re[k,t]

}

## Distance sampling probability estimation using summation technique: Pr(p of x)=exp(-x^2/2*sigma^2)*f(x)

for(b in 1:n.breaks){

log(g[b,k])<- -mdpts[b]*mdpts[b]/(2*sigma[k]*sigma[k]) # half-normal detection function - first half of eq.,

f[b,k]<- ( 2*mdpts[b]*delta )/(maxd*maxd) # this is f(x), the scaled radial density function (distribution of area around point)

# need [b] index on delta if distance intervals are not all of equal size

pi.pd[b,k]<- g[b,k]*f[b,k] #this is the product Pr(detect)*Pr(distribution)

pi.pd.c[b,k]<- pi.pd[b,k]/pdet[k] # standardizing based on overall capture probability - conditional formulation

}

pdet[k]<-sum(pi.pd[,k]) # probability of detection is the sum of all the rectangular areas calculated above

}

### model likelihood

## Observation-level model

for(i in 1:n.obs){

#single binomial trial with categorical distribution linking distance class and time interval to survey point

dclass[i] ~ dcat(pi.pd.c[,obs.surveyid[i]])

}

## Abundance estimation

for(k in 1:n.surveys){

# site abundance model

log(lambda[k])<- beta.int + incl.mangrove*beta.mangrove*mangrove[k] + incl.all_land*beta.all_land*all_land[k] +

incl.seagrass*beta.seagrass2009*seagrass2009[k]*(1-seagrass2015[k]) + incl.seagrass*beta.seagrass2015*seagrass2015[k] +

beta.D.occupied*D.occupied[k] + survey.re[k]

N[k]~dpois(lambda[k])# predicted abundance per point

# loop through surveys

for(j in 1:n.rep.counts){

# binomial model for # of captured individuals

y[k,j]~ dbin(pdet[k]*pavail[k,j],N[k]) # counts related to probability of detection, given availability

}

}

}", fill=TRUE)

sink()

# run full model ----------------------------------------------------------

library(jagsUI)

library(tidyverse)

library(coda)

library(ggmcmc)

library(bayesplot)

load("./RData/observed terrapin data final.RData")

Nst<-apply(terrapin.obs.data$y,1,max)

inits.expanded <- function(){list(N=Nst+1,navail=Nst+1, sigma.int=runif(1,1.6,5.7),beta.int=runif(1,0,5),

beta.mangrove=runif(1,-2,2), beta.all_land=0, beta.D.occupied=runif(1,-2,2),

beta.seagrass2009=runif(1,-2,2), beta.seagrass2015=runif(1,-2,2),

z=rep(1,nrow(terrapin.obs.data$y))

)}

params.OD <- c("beta.int", "beta.mangrove", "beta.all_land", "beta.seagrass2009", "beta.seagrass2015",

"beta.D.occupied", "beta.a0", "sigma.int", "survey.sd", "OD.sd",

"BPV", "fit.obs", "fit.new")

out.OD.surveyRE <- jags(data=terrapin.obs.data, inits=inits.expanded, parameters.to.save=params.OD,

model.file="./BUGS/terrapin__final_OD_surveyRE.txt",

n.chains=3, n.adapt=NULL, n.thin=1, parallel=T, n.iter=50000, n.burnin = 25000)

print(out.OD.surveyRE, digits=3)

out.OD.surveyRE.update <- update(out.OD.surveyRE, n.iter = 200000)

print(out.OD.surveyRE.update, digits=3)

plot(out.OD.surveyRE.update$sims.list$fit.obs, out.OD.surveyRE.update$sims.list$fit.new);abline(0,1,col="red")

out.jags.mcmc <- as.mcmc.list(out.OD.surveyRE.update$samples)

out.jags.mcmc.thin <- window(out.jags.mcmc, thin=100)

S <- ggs(out.jags.mcmc.thin)

ggmcmc(S, file="./output/second paper/survey/ODfullmodel_plot1.pdf", plot=c("density", "traceplot", "running"))

ggmcmc(S, file="./output/second paper/survey/ODfullmodel_plot2.pdf", plot=c("compare_partial", "autocorrelation", "crosscorrelation", "Rhat", "geweke", "caterpillar"))

# check residuals

params.resids <- c("TF.stat", "TF.stat.new")

out.OD.surveyRE.update.resids <- update(out.OD.surveyRE.update, n.iter = 20000, parameters.to.save = params.resids)

newid.alias <- data.frame(newid=sort(unique(sample.covariate.data$newid)),

index =seq_along(sample.covariate.data$newid))

resids <- cbind(newid.alias,

TF.stat=apply(out.OD.surveyRE.update.resids$mean$TF.stat,1,sum),

TF.stat.new=apply(out.OD.surveyRE.update.resids$mean$TF.stat.new,1,sum),

terrapin.obs.data$y)

resids$diff <- resids$TF.stat-resids$TF.stat.new

plot(jitter(resids$diff), ylim=c(-1,1)); abline(h=0)

Prior.params <- out.OD.surveyRE.update$summary[2:6,1:2]

# save.image("./RData/terrapin_data_full.RData")

# run MS ------------------------------------------------------------------

slab.priors <- list(mu.mangrove = c(Prior.params["beta.mangrove",1],0),

mu.all.land = c(Prior.params["beta.all_land",1],0),

mu.seagrass2009 = c(Prior.params["beta.seagrass2009",1],0),

mu.seagrass2015 = c(Prior.params["beta.seagrass2015",1],0),

mu.D.occupied = c(Prior.params["beta.D.occupied",1],0),

tau.mangrove = c(1/Prior.params["beta.mangrove",2],0.1),

tau.all.land = c(1/Prior.params["beta.all_land",2],0.1),

tau.seagrass2009 = c(1/Prior.params["beta.seagrass2009",2],0.1),

tau.seagrass2015 = c(1/Prior.params["beta.seagrass2015",2],0.1),

tau.D.occupied = c(1/Prior.params["beta.D.occupied",2],0.1)

)

Nst<-apply(terrapin.obs.data$y,1,max)

inits <- function(){list(N=Nst+1,navail=Nst+1, sigma.int=runif(1,1.6,300),beta.int=runif(1,0,5),

beta.mangrove=runif(1,-2,2), beta.all_land=0, beta.D.occupied=runif(1,-2,2),

beta.seagrass2009=runif(1,-2,2), beta.seagrass2015=runif(1,-2,2)

)}

# parameters to estimate

params<-c("beta.int", "beta.mangrove", "beta.all_land", "beta.seagrass2009", "beta.seagrass2015",

"beta.D.occupied", "ms.mangrove", "ms.all_land", "ms.seagrass", "ms.D.occupied",

"beta.a0", "sigma.int", "survey.sd", "OD.sd", "BPV")

out.MS <- autojags(data=c(terrapin.obs.data, slab.priors), inits=inits, parameters.to.save=params,

model.file="./BUGS/Observed_terrapin__MS.txt",

n.chains=3, n.adapt=NULL, n.thin=1, parallel=T,

Rhat.limit=1.01, max.iter=2000000, iter.increment=50000)

print(out.MS,dig=3)

# save.image("./RData/model selection.RData")

out.jags.mcmc <- as.mcmc.list(out.MS$samples)

out.jags.mcmc.thin <- window(out.jags.mcmc, thin=100)

S <- ggs(out.jags.mcmc.thin)

ggmcmc(S, file="./output/second paper/survey/MS_BI_plot1.pdf", plot=c("density", "traceplot", "running"))

ggmcmc(S, file="./output/second paper/survey/MS_BI_plot2.pdf", plot=c("compare_partial", "autocorrelation", "crosscorrelation", "Rhat", "geweke", "caterpillar"))

out.MS.update <- update(out.MS, parameters.to.save = params, n.iter=500000)

print(out.MS.update,dig=3)

# save.image("./RData/model selection.RData")

out.jags.mcmc <- as.mcmc.list(out.MS.update$samples)

out.jags.mcmc.thin <- window(out.jags.mcmc, thin=100)

S <- ggs(out.jags.mcmc.thin)

ggmcmc(S, file="./output/second paper/survey/MS_plot1.pdf", plot=c("density", "traceplot", "running"))

ggmcmc(S, file="./output/second paper/survey/MS_plot2.pdf", plot=c("compare_partial", "autocorrelation", "crosscorrelation", "Rhat", "geweke", "caterpillar"))

out.MS.update.2 <- update(out.MS.update, parameters.to.save = params, n.iter=500000)

print(out.MS.update.2,dig=3)

out.MS.update.3 <- update(out.MS.update.2, parameters.to.save = params, n.iter=1000000)

print(out.MS.update.3,dig=3)

out.MS.update.4 <- update(out.MS.update.3, parameters.to.save = params, n.iter=1000000)

print(out.MS.update.4,dig=3)

# combine runs

library(runjags)

source("C:/Users/estolen/OneDrive - NASA/Documents/Stats/R/EDSfunctions/combine mcmc function.R")

comb.mcmc <- combine.mcmc(list(out.MS.update$samples,

out.MS.update.2$samples,

out.MS.update.3$samples,

out.MS.update.4$samples))

summary.tbl <- posterior.tbl_eds(comb.mcmc, type="list")

# summary.tbl <- summary(comb.mcmc)

# size.tbl <- effectiveSize(Hybrid.TerrSite.gof$samples)

# GelRub <- gelman.diag(Hybrid.TerrSite.gof$samples, transform = T)

# (out.tbl <- cbind(summary.tbl$statistics[,1:2],summary.tbl$quantiles[,c(1,3,5)],

# Rhat=round(GelRub$psrf[,1],2), n.eff=round(size.tbl)))

#

write.csv(summary.tbl, "./output/second paper/survey/Posterior_summary.csv")

# Extract the indicator variables for model selection

comb.mcmc.collapsed <- combine.mcmc(list(out.MS.update$samples,

out.MS.update.2$samples,

out.MS.update.3$samples,

out.MS.update.4$samples),

collapse.chains = T)

str(comb.mcmc.collapsed)

temp.att <- attributes(comb.mcmc.collapsed)$dimnames[[2]]

temp.mcmc <- comb.mcmc

w.ms.all_land <- comb.mcmc.collapsed[,which(temp== "ms.all_land")]

w.ms.mangrove <- comb.mcmc.collapsed[,which(temp== "ms.mangrove")]

w.ms.seagrass <- comb.mcmc.collapsed[,which(temp== "ms.seagrass")]

w.ms.D.occupied <- comb.mcmc.collapsed[,which(temp== "ms.D.occupied")]

# Combine into a model indicator string and tabulate posterior frequencies

mod <- paste(w.ms.all_land, w.ms.mangrove,w.ms.seagrass,w.ms.D.occupied)

model.tb <- as.data.frame(round(table(mod)/length(w.ms.all_land),5))

variables <- c("all land", "mangrove", "seagrass", "D.occupied")

model.tb$model <- NA

for(i in 1:dim(model.tb)[1]){

index <- which(as.numeric(unlist(strsplit(as.character(model.tb[i,1]),split = " ")))==1)

model.tb[i,"model"] <- paste(variables[index], collapse=" + ")

}

model.tb[order(model.tb$Freq, decreasing = T),]

write.csv(model.tb, file="./output/second paper/survey/model_slection_table.csv")

model.tb[order(model.tb$Freq, decreasing = T),]

write.csv(summary.tbl, "./output/second paper/survey/Posterior_summary.csv")

save.image("./RData/model selection.RData")

# plot(out.OD.surveyRE.update$sims.list$fit.obs, out.OD.surveyRE.update$sims.list$fit.new);abline(0,1,col="red")

# run beta checks ------------------------------------------------------------------

Nst<-apply(terrapin.obs.data$y,1,max)

inits <- function(){list(N=Nst+1,navail=Nst+1, sigma.int=runif(1,1.6,300),beta.int=runif(1,0,5),

beta.mangrove=runif(1,-2,2), beta.all_land=0,

beta.seagrass2009=runif(1,-2,2), beta.seagrass2015=runif(1,-2,2),

beta.D.occupied=runif(1,-2,2)

)}

# parameters to estimate

params.beta <- c("beta.int", "beta.mangrove", "beta.all_land", "beta.seagrass2009", "beta.seagrass2015",

"beta.D.occupied", "beta.a0", "sigma.int", "survey.sd", "OD.sd")

incl.all_land <- list(incl.mangrove=0, incl.all_land=1, incl.seagrass=0, incl.D.occupied=0)

incl.mang <- list(incl.mangrove=1, incl.all_land=0, incl.seagrass=0, incl.D.occupied=0)

incl.seagrass <- list(incl.mangrove=0, incl.all_land=0, incl.seagrass=1, incl.D.occupied=0)

incl.D.occupied <- list(incl.mangrove=0, incl.all_land=0, incl.seagrass=0, incl.D.occupied=1)

incl.all <- list(incl.mangrove=1, incl.all_land=1, incl.seagrass=1, incl.D.occupied=1)

# all.land

out.incl.all_land <- autojags(data=c(terrapin.obs.data, incl.all_land), inits=inits,

parameters.to.save=params.beta,

model.file="./BUGS/Observed_terrapin__betas.txt",

n.chains=3, n.adapt=NULL, n.thin=1, parallel=T,

Rhat.limit=1.05, max.iter=2000000, iter.increment=50000)

print(out.incl.all_land,dig=3)

out.incl.all_land.update <- update(out.incl.all_land, n.iter=100000)

print(out.incl.all_land.update,dig=3)

# mangrove

out.incl.mang <- autojags(data=c(terrapin.obs.data, incl.mang), inits=inits,

parameters.to.save=params.beta,

model.file="./BUGS/Observed_terrapin__betas.txt",

n.chains=3, n.adapt=NULL, n.thin=1, parallel=T,

Rhat.limit=1.05, max.iter=2000000, iter.increment=50000)

print(out.incl.mang,dig=3)

out.incl.mang.update <- update(out.incl.mang, n.iter=100000)

print(out.incl.mang.update,dig=3)

# sea grass

out.incl.seagrass <- autojags(data=c(terrapin.obs.data, incl.seagrass), inits=inits,

parameters.to.save=params.beta,

model.file="./BUGS/Observed_terrapin__betas.txt",

n.chains=3, n.adapt=NULL, n.thin=1, parallel=T,

Rhat.limit=1.05, max.iter=2000000, iter.increment=50000)

print(out.incl.seagrass,dig=3)

out.incl.seagrass.update <- update(out.incl.seagrass, n.iter=100000)

print(out.incl.seagrass.update,dig=3)

# D.occupied

out.incl.D.occupied <- autojags(data=c(terrapin.obs.data, incl.D.occupied), inits=inits,

parameters.to.save=params.beta,

model.file="./BUGS/Observed_terrapin__betas.txt",

n.chains=3, n.adapt=NULL, n.thin=1, parallel=T,

Rhat.limit=1.05, max.iter=2000000, iter.increment=50000)

print(out.incl.D.occupied,dig=3)

out.incl.D.occupied.update <- update(out.incl.D.occupied, n.iter=100000)

print(out.incl.D.occupied.update,dig=3)

# all

out.incl.incl.all <- autojags(data=c(terrapin.obs.data, incl.all), inits=inits,

parameters.to.save=params.beta,

model.file="./BUGS/Observed_terrapin__betas.txt",

n.chains=3, n.adapt=NULL, n.thin=1, parallel=T,

Rhat.limit=1.05, max.iter=2000000, iter.increment=50000)

print(out.incl.incl.all,dig=3)

out.incl.incl.all.update <- update(out.incl.incl.all, n.iter=100000)

print(out.incl.incl.all.update,dig=3)

# compare betas

sink("./output/betas.txt")

out.incl.incl.all.update$summary

out.incl.all_land.update$summary

out.incl.mang.update$summary

out.incl.seagrass.update$summary

out.incl.D.occupied.update$summary

sink()

save.image("./RData/model selection.RData")

# explore covariates ------------------------------------------------------

str(terrapin.obs.data)

terrapin.cov <- as.data.frame(terrapin.obs.data[13:17])

pairs(terrapin.cov)

with(terrapin.cov,plot(mangrove~all_land))

with(terrapin.cov,plot(as.factor(seagrass2009)~as.factor(seagrass2015)))

xtabs(~seagrass2009+seagrass2015,terrapin.cov)

# 94 no sea grass either year, 53 sea grass 2009 only, 18 sea grass 2009 and 2015

with(terrapin.cov,plot(mangrove~Died))

with(terrapin.cov,plot(Died~all_land))

cor(terrapin.cov)

# run all land + sea grass ----------------------------------------------------

Nst<-apply(terrapin.obs.data$y,1,max)

inits <- function(){list(N=Nst+1,navail=Nst+1, sigma.int=runif(1,0.48,5.7),beta.int=runif(1,0,5),

beta.mangrove=runif(1,-2,2), beta.all_land=0,

beta.seagrass2009=runif(1,-2,2), beta.seagrass2015=runif(1,-2,2),

beta.Died=runif(1,-2,2))}

# parameters to estimate

params <- c("beta.int", "beta.mangrove", "beta.all_land", "beta.seagrass2009", "beta.seagrass2015",

"meanpdet", "meanN", "labmbda.int", "avail.int",

"beta.a0", "sigma.int")

incl.all_land_seagrass <- list(incl.mangrove=0, incl.all_land=1, incl.seagrass=1)

# all.land

out.all_land_seagrass <- autojags(data=c(terrapin.obs.data, incl.all_land_seagrass), inits=inits,

parameters.to.save=params.beta,

model.file="./BUGS/Observed_terrapin__betas.txt",

n.chains=3, n.adapt=NULL, n.thin=1, parallel=T,

Rhat.limit=1.05, max.iter=2000000, iter.increment=50000)

print(out.all_land_seagrass,dig=3)

out.all_land_seagrass.update <- update(out.all_land_seagrass, n.iter=250000)

print(out.all_land_seagrass.update,dig=3)

out.jags.mcmc <- as.mcmc.list(out.all_land_seagrass.update$samples)

out.jags.mcmc.thin <- window(out.jags.mcmc, thin=100)

S <- ggs(out.jags.mcmc.thin)

ggmcmc(S, file="./output/second paper/survey/all_land_seagrass_plot1.pdf", plot=c("density", "traceplot", "running"))

ggmcmc(S, file="./output/second paper/survey/all_land_seagrass_plot2.pdf", plot=c("compare_partial", "autocorrelation", "crosscorrelation", "Rhat", "geweke", "caterpillar"))

# plot causal DAG ---------------------------------------------------------

library(dagitty)

g1 <- dagitty("dag{

L -> N ;

L -> SG -> N ;

L -> M -> N

}")

g2 <- dagitty("dag{

L -> N ;

L -> SG -> N ;

L -> M -> N;

L -> O -> N;

L -> SG -> O -> N ;

L -> M -> O -> N

}")

coordinates(g1) <- list(x=c(L=-10, M=-5, SG=10, N=5),

y=c(L=0, M=-10, SG=0, N=10))

plot(graphLayout(g1))

coordinates(g2) <- list(x=c(L=-10, M=-5, SG=10, N=5, O=15 ),

y=c(L=0, M=-10, SG=0, N=10, O=10 ))

plot(graphLayout(g2))

print( impliedConditionalIndependencies( g1 ) )

print( impliedConditionalIndependencies( g2 ) )

save.image("./RData/modelselection seagrass combined.RData")

# estimate density --------------------------------------------------------

all.equal(terrapin.obs.data$mangrove*attributes.ls$Mangrove$`scaled:scale` + attributes.ls$Mangrove$`scaled:center`,

sample.covariate.data$mang)

summary(sample.covariate.data)

hist(sample.covariate.data$all_land,40)

fivenum(sample.covariate.data$all_land)

fivenum(terrapin.obs.data$all_land)

(c(75, 115, 215)-attributes.ls$all_land$`scaled:center`)/attributes.ls$all_land$`scaled:scale`

# comb.mcmc <- combine.mcmc(list(out.MS.update$samples,

# out.MS.update.2$samples,

# out.MS.update.3$samples,

# out.MS.update.4$samples))

# summary.tbl <- posterior.tbl_eds(comb.mcmc, type="list")

# comb.mcmc.collapsed <- combine.mcmc(list(out.MS.update$samples,

# out.MS.update.2$samples,

# out.MS.update.3$samples,

# out.MS.update.4$samples),

# collapse.chains = T)

comb.mcmc.collapsed

# calculate density at 3 levels of all_land (lower hinge, median, upper hing)

temp.att <- attributes(comb.mcmc.collapsed)$dimnames[[2]]

density.est.lq <- exp(comb.mcmc.collapsed[,which(temp.att=="beta.int")] + comb.mcmc.collapsed[,which(temp.att=="beta.all_land")]*fivenum(terrapin.obs.data$all_land)[2])

density.est.med <- exp(comb.mcmc.collapsed[,which(temp.att=="beta.int")] + comb.mcmc.collapsed[,which(temp.att=="beta.all_land")]*fivenum(terrapin.obs.data$all_land)[3])

density.est.uq <- exp(comb.mcmc.collapsed[,which(temp.att=="beta.int")] + comb.mcmc.collapsed[,which(temp.att=="beta.all_land")]*fivenum(terrapin.obs.data$all_land)[4])

mean(comb.mcmc.collapsed[,which(temp.att=="beta.int")])

mean(exp(comb.mcmc.collapsed[,which(temp.att=="beta.int")]))

plot.area <- pi*75^2/10000

mean(density.est.lq)/plot.area

mean(density.est.med)/plot.area

mean(density.est.uq)/plot.area

median(density.est.lq)/plot.area

median(density.est.med)/plot.area

median(density.est.uq)/plot.area

exp(-1.66-2.77*fivenum(terrapin.obs.data$all_land))/plot.area

density.est.lq <- exp(comb.mcmc.collapsed[,which(temp.att=="beta.int")] + comb.mcmc.collapsed[,which(temp.att=="beta.seagrass2015")] + comb.mcmc.collapsed[,which(temp.att=="beta.all_land")]*fivenum(terrapin.obs.data$all_land)[2])

density.est.med <- exp(comb.mcmc.collapsed[,which(temp.att=="beta.int")] + comb.mcmc.collapsed[,which(temp.att=="beta.seagrass2015")] + comb.mcmc.collapsed[,which(temp.att=="beta.all_land")]*fivenum(terrapin.obs.data$all_land)[3])

density.est.uq <- exp(comb.mcmc.collapsed[,which(temp.att=="beta.int")] + comb.mcmc.collapsed[,which(temp.att=="beta.seagrass2015")] + comb.mcmc.collapsed[,which(temp.att=="beta.all_land")]*fivenum(terrapin.obs.data$all_land)[4])

mean(comb.mcmc.collapsed[,which(temp.att=="beta.int")])

mean(exp(comb.mcmc.collapsed[,which(temp.att=="beta.int")]))

plot.area <- pi*75^2/10000

mean(density.est.lq)/plot.area

mean(density.est.med)/plot.area

mean(density.est.uq)/plot.area

median(density.est.lq)/plot.area

median(density.est.med)/plot.area

median(density.est.uq)/plot.area

exp(-1.66-2.77*fivenum(terrapin.obs.data$all_land))/plot.area

**Supplemental 6. R instructions to run full model for**

**terrapin survey data**

# full BUGS model --------------------------------------------------------------

sink("./BUGS/terrapin__final_OD_surveyRE.txt")

cat("

model {

## PRIORS

# priors for distance function

# sigma.int ~ dunif(0.48,5.7)

sigma.int ~ dunif(1.6,300) # intercept for sigma in Distance function (Sigma cannot be <1.6 or det=0 for some diatnaces)

# priors for availability covariate model

beta.a0 ~ dbeta(1,1)

beta.a0.logit <- logit(beta.a0)

survey.sd ~ dnorm(0, 0.2)I(0,) # hyperprior for RE sd

survey.tau <- 1 / (survey.sd * survey.sd)

for(p in 1:n.surveys){

survey.re[p] ~ dnorm(0,survey.tau)

}

OD.sd ~ dnorm(0, 0.2)I(0,) # hyperprior for OD RE sd

OD.tau <- 1 / (OD.sd * OD.sd)

for(p in 1:n.surveys){

for(t in 1:n.rep.counts){

OD.re[p,t] ~ dnorm(0,OD.tau)

}

}

beta.int ~ dt(0,1/(2.5^2),7)

beta.all_land ~ dt(0,1/(2.5^2),7)

beta.mangrove ~ dt(0,1/(2.5^2),7)

beta.seagrass2009 ~ dt(0,1/(2.5^2),7)

beta.seagrass2015 ~ dt(0,1/(2.5^2),7)

beta.D.occupied ~ dt(0,1/(2.5^2),7)

## Detection functions (Detection = availability + perceptibility)

for(k in 1:n.surveys){

# covariates for scale parameter of Distance function (perceptibility)

log(sigma[k]) <- (sigma.int)

# covariates for availability parameters of Tme-Removal (availability)

for(t in 1:n.rep.counts){

logit(pavail[k,t]) <- beta.a0.logit + OD.re[k,t]

}

## Distance sampling probability estimation using summation technique: Pr(p of x)=exp(-x^2/2*sigma^2)*f(x)

for(b in 1:n.breaks){

log(g[b,k])<- -mdpts[b]*mdpts[b]/(2*sigma[k]*sigma[k]) # half-normal detection function - first half of eq.,

f[b,k]<- ( 2*mdpts[b]*delta )/(maxd*maxd) # this is f(x), the scaled radial density function (distribution of area around point)

# need [b] index on delta if distance intervals are not all of equal size

pi.pd[b,k]<- g[b,k]*f[b,k] #this is the product Pr(detect)*Pr(distribution)

pi.pd.c[b,k]<- pi.pd[b,k]/pdet[k] # standardizing based on overall capture probability - conditional formulation

}

pdet[k]<-sum(pi.pd[,k]) # probability of detection is the sum of all the rectangular areas calculated above

}

### model likelihood

## Observation-level model

for(i in 1:n.obs){

dclass[i] ~ dcat(pi.pd.c[,obs.surveyid[i]])

}

## Abundance estimation

for(k in 1:n.surveys){

# site abundance model

log(lambda[k])<- beta.int + beta.mangrove*mangrove[k] + beta.all_land*all_land[k] +

beta.seagrass2009*seagrass2009[k]*(1-seagrass2015[k]) + beta.seagrass2015*seagrass2015[k] +

beta.D.occupied*D.occupied[k] + survey.re[k]

N[k]~dpois(lambda[k])# predicted abundance per point

# loop through surveys

for(j in 1:n.rep.counts){

# binomial model for # of captured individuals

y[k,j]~ dbin(pdet[k]*pavail[k,j],N[k])

y.new[k,j] ~ dbin(pdet[k]*pavail[k,j],N[k])

y.expected[k,j] <- pdet[k]*pavail[k,j]*N[k]

TF.stat[k,j] <- pow(sqrt(y[k,j]) - sqrt( y.expected[k,j]),2)

TF.stat.new[k,j] <- pow(sqrt(y.new[k,j]) - sqrt(y.expected[k,j]),2)

}

}

## posterior predictive check (Bayesian p value, BPV)

fit.obs<- sum(TF.stat[,])

fit.new<- sum(TF.stat.new[,])

BPV <- (fit.obs < fit.new)

}", fill=TRUE)

sink()

# run full model ----------------------------------------------------------

library(jagsUI)

library(tidyverse)

library(coda)

library(ggmcmc)

library(bayesplot)

load("./RData/observed terrapin data final.RData")

Nst<-apply(terrapin.obs.data$y,1,max)

inits.expanded <- function(){list(N=Nst+1,navail=Nst+1, sigma.int=runif(1,1.6,5.7),beta.int=runif(1,0,5),

beta.mangrove=runif(1,-2,2), beta.all_land=0, beta.D.occupied=runif(1,-2,2),

beta.seagrass2009=runif(1,-2,2), beta.seagrass2015=runif(1,-2,2),

z=rep(1,nrow(terrapin.obs.data$y))

)}

params.OD <- c("beta.int", "beta.mangrove", "beta.all_land", "beta.seagrass2009", "beta.seagrass2015",

"beta.D.occupied", "beta.a0", "sigma.int", "survey.sd", "OD.sd",

"BPV", "fit.obs", "fit.new")

out.OD.surveyRE <- autojags(data=terrapin.obs.data, inits=inits.expanded, parameters.to.save=params.OD,

model.file="./BUGS/terrapin__final_OD_surveyRE.txt",

n.chains=3, n.adapt=NULL, n.thin=1, parallel=T,

iter.increment = 50000, n.burnin = 50000, Rhat.limit=1.01,

max.iter=500000)

print(out.OD.surveyRE, digits=3)

out.jags.mcmc <- as.mcmc.list(out.OD.surveyRE$samples)

out.jags.mcmc.thin <- window(out.jags.mcmc, thin=100)

S <- ggs(out.jags.mcmc.thin)

ggmcmc(S, file="./output/second paper/survey/ODfullmodel_BI_plot1.pdf", plot=c("density", "traceplot", "running"))

ggmcmc(S, file="./output/second paper/survey/ODfullmodel_BI_plot2.pdf", plot=c("compare_partial", "autocorrelation", "crosscorrelation", "Rhat", "geweke", "caterpillar"))

out.OD.surveyRE.update <- update(out.OD.surveyRE, n.iter = 1000000)

print(out.OD.surveyRE.update, digits=3)

out.OD.surveyRE.update.2 <- update(out.OD.surveyRE.update, n.iter = 1000000)

print(out.OD.surveyRE.update.2, digits=3)

library(runjags)

source("C:/Users/estolen/OneDrive - NASA/Documents/Stats/R/EDSfunctions/combine mcmc function.R")

comb.mcmc <- combine.mcmc(list(out.OD.surveyRE.update$samples, out.OD.surveyRE.update.2$samples))

summary.tbl <- posterior.tbl_eds(comb.mcmc, type="list")

write.csv(summary.tbl, file="./output/second paper/survey/posterier estimates full model no MS.csv")

comb.mcmc.collapsed <- combine.mcmc(list(out.OD.surveyRE.update$samples, out.OD.surveyRE.update.2$samples), collapse.chains = T)

subset <- sample(dim(comb.mcmc.collapsed)[1],10000)

plot(comb.mcmc.collapsed[subset,12], comb.mcmc.collapsed[subset,13]);abline(0,1,col="red")

# out.jags.mcmc <- as.mcmc.list(out.OD.surveyRE.update$samples)

out.jags.mcmc.thin <- window(comb.mcmc, thin=10000)

S <- ggs(out.jags.mcmc.thin)

ggmcmc(S, file="./output/second paper/survey/ODfullmodel_all_plot1.pdf", plot=c("density", "traceplot", "running"))

ggmcmc(S, file="./output/second paper/survey/ODfullmodel_all_plot2.pdf", plot=c("compare_partial", "autocorrelation", "crosscorrelation", "Rhat", "geweke", "caterpillar"))

# save.image("./RData/terrapin_data_final_noMS.RData")

# check residuals

params.resids <- c("TF.stat", "TF.stat.new")

out.OD.surveyRE.update.resids <- update(out.OD.surveyRE.update.2, n.iter = 20000, parameters.to.save = params.resids)

newid.alias <- data.frame(newid=sort(unique(sample.covariate.data$newid)),

index =seq_along(sample.covariate.data$newid))

resids <- cbind(newid.alias,

TF.stat=apply(out.OD.surveyRE.update.resids$mean$TF.stat,1,sum),

TF.stat.new=apply(out.OD.surveyRE.update.resids$mean$TF.stat.new,1,sum),

terrapin.obs.data$y)

resids$diff <- resids$TF.stat-resids$TF.stat.new

plot(jitter(resids$diff), ylim=c(-1,1)); abline(h=0)

save.image("./RData/terrapin_data_final_noMS.RData")

# Prior.params <- out.OD.surveyRE.update$summary[2:6,1:2]

# save.image("./RData/terrapin_data_full.RData")

# plot posteriors ---------------------------------------------------------

full_noMS_comb.mcmc <- combine.mcmc(list(out.OD.surveyRE.update$samples, out.OD.surveyRE.update.2$samples),

collapse.chains = T)

attributes(full_noMS_comb.mcmc)

attributes(full_noMS_comb.mcmc)$dimnames[[2]]

color_scheme_set(scheme="gray")

(plot.post.bar <- mcmc_intervals(full_noMS_comb.mcmc,

pars=c("beta.int", "beta.mangrove", "beta.all_land", "beta.seagrass2009",

"beta.seagrass2015", "beta.D.occupied", "beta.a0", "sigma.int",

"survey.sd", "OD.sd"), outer_size = 0.5, inner_size = 1,

point_size = 2) +

geom_vline(xintercept=0) + theme(axis.text = element_text(size = 11))

)

ppi=300

tiff(file="./output/second paper/survey/full model no MS posterior bar medres.tiff",

width = 6*ppi, height = 6*ppi, res = ppi)

plot.post.bar

dev.off()
